# Lysosomal TRPML1 triggers global Ca^2+^ signals and nitric oxide release in human cerebrovascular endothelial cells

**DOI:** 10.3389/fphys.2024.1426783

**Published:** 2024-06-21

**Authors:** Valentina Brunetti, Roberto Berra-Romani, Filippo Conca, Teresa Soda, Gerardo Rosario Biella, Andrea Gerbino, Francesco Moccia, Giorgia Scarpellino

**Affiliations:** ^1^ Laboratory of General Physiology, Department of Biology and Biotechnology “L. Spallanzani”, University of Pavia, Pavia, Italy; ^2^ Department of Biomedicine, School of Medicine, Benemérita Universidad Autónoma de Puebla, Puebla, Mexico; ^3^ Department of Molecular Medicine, University of Pavia, Pavia, Italy; ^4^ Veneto Institute of Molecular Medicine, Foundation for Advanced Biomedical Research, Padova, Italy; ^5^ Department of Health Sciences, University of Magna Graecia, Catanzaro, Italy; ^6^ Department of Biosciences, Biotechnologies and Biopharmaceutics, University of Bari “Aldo Moro”, Bari, Italy; ^7^ Department of Medicine and Health Sciences “V. Tiberio”, University of Molise, Campobasso, Italy

**Keywords:** TRPML1, lysosomes, Ca^2+^ signaling, nitric oxide, endothelial cells, InsP3 receptors, store-operated Ca^2+^ entry

## Abstract

Lysosomal Ca^2+^ signaling is emerging as a crucial regulator of endothelial Ca^2+^ dynamics. Ca^2+^ release from the acidic vesicles in response to extracellular stimulation is usually promoted *via* Two Pore Channels (TPCs) and is amplified by endoplasmic reticulum (ER)-embedded inositol-1,3,4-trisphosphate (InsP_3_) receptors and ryanodine receptors. Emerging evidence suggests that sub-cellular Ca^2+^ signals in vascular endothelial cells can also be generated by the Transient Receptor Potential Mucolipin 1 channel (TRPML1) channel, which controls vesicle trafficking, autophagy and gene expression. Herein, we adopted a multidisciplinary approach, including live cell imaging, pharmacological manipulation, and gene targeting, revealing that TRPML1 protein is expressed and triggers global Ca^2+^ signals in the human brain microvascular endothelial cell line, hCMEC/D3. The direct stimulation of TRPML1 with both the synthetic agonist, ML-SA1, and the endogenous ligand phosphatidylinositol 3,5-bisphosphate (PI(3,5)P_2_) induced a significant increase in [Ca^2+^]_i,_ that was reduced by pharmacological blockade and genetic silencing of TRPML1. In addition, TRPML1-mediated lysosomal Ca^2+^ release was sustained both by lysosomal Ca^2+^ release and ER Ca^2+^- release through inositol-1,4,5-trisphophate receptors and store-operated Ca^2+^ entry. Notably, interfering with TRPML1-mediated lysosomal Ca^2+^ mobilization led to a decrease in the free ER Ca^2+^ concentration. Imaging of DAF-FM fluorescence revealed that TRPML1 stimulation could also induce a significant Ca^2+^-dependent increase in nitric oxide concentration. Finally, the pharmacological and genetic blockade of TRPML1 impaired ATP-induced intracellular Ca^2+^ release and NO production. These findings, therefore, shed novel light on the mechanisms whereby the lysosomal Ca^2+^ store can shape endothelial Ca^2+^ signaling and Ca^2+^-dependent functions in vascular endothelial cells.

## 1 Introduction

An increase in intracellular Ca^2+^ concentration ([Ca^2+^]_i_) with different spatio-temporal features is the primary mechanism by which vascular endothelial cells and endothelial colony forming cells (ECFCs) regulate a plethora of cardiovascular functions (McCarron et al., 2017; Moccia et al., 2023b), including vascular tone and permeability (De Bock et al., 2013; Ottolini et al., 2019), angiogenesis (Moccia et al., 2019; Negri et al., 2019), and vasculogenesis (Lodola et al., 2017; Zuccolo et al., 2018). The endothelial Ca^2+^ response to extracellular stimuli, including hormones, neurotransmitters, and growth factors, is triggered by inositol-1,4,5-trisphosphate (InsP_3_)-induced Ca^2+^ release from the endoplasmic reticulum (ER) and maintained over time by store-operated Ca^2+^ entry (SOCE) (Moccia et al., 2023a; Moccia et al., 2023b). However, recent studies have highlighted that the lysosomal Ca^2+^ store can also play a crucial role in patterning the endothelial Ca^2+^ signals (Moccia et al., 2021a; Negri et al., 2021b). The second messenger nicotinic acid adenine dinucleotide phosphate (NAADP) can support intracellular Ca^2+^ release by gating two pore channels (TPCs), which exist in two isoforms and mediate lysosomal Ca^2+^ release in response to several endothelial autacoids (Brailoiu et al., 2010; Esposito et al., 2011; Favia et al., 2014; Zuccolo et al., 2019a; Zuccolo et al., 2019b; Berra-Romani et al., 2020; Negri et al., 2022). Transient Receptor Potential Mucolipin 1 (TRPML1) provides an additional pathway for lysosomal Ca^2+^ release (Kilpatrick et al., 2016; Thakore et al., 2020; Negri et al., 2021b; Moccia et al., 2023c; Scorza et al., 2023). TRPML1 is mainly located in late endosomes and lysosomes (Dong et al., 2010; Zhang et al., 2012; Kilpatrick et al., 2016; Li et al., 2019) and may be gated by the lysosome-associated phosphoinositide, phosphatidylinositol 3,5-bisphosphate, (PI(3,5)P_2_) (Dong et al., 2010; Zhang et al., 2012), which is generated by the lipid kinase PYKfive (Isobe et al., 2019). It has been widely recognized that TRPML1 mediates local Ca^2+^ signals that normally do not spread to the whole cytosol, but are spatially restricted to the perilysosomal space to control autophagy (Medina et al., 2015), lysosome volume (Cao et al., 2017), and lysosome trafficking (Xu and Ren, 2015). However, several studies have recently shown that TRPML1-mediated lysosomal Ca^2+^ release may also result in global increases in [Ca^2+^]_i_ that require the functional interaction with ER Ca^2+^ release through InsP_3_Rs or ryanodine receptors (RyRs) and with SOCE (Kilpatrick et al., 2016; Thakore et al., 2020; Boretto et al., 2023; Scorza et al., 2023). Surprisingly, little information is available about the role played by TRPML1 in vascular endothelial cells (Negri et al., 2021b; Lai et al., 2024; Yang et al., 2024; Zhang et al., 2024). The synthetic TRPML1 agonist, ML-SA1, induces peri-lysosomal Ca^2+^ signals that promote lysosome movement and lysosome-multivesicular bodies interaction in mouse coronary artery endothelial cells (Li et al., 2022). It is, however, still unknown whether TRPML1 also induces Ca^2+^ signals in human vascular endothelial cells and whether they are supported by InsP_3_-induced ER Ca^2+^ release.

The hCMEC/D3 cell line is the most widely used model of human cerebrovascular endothelial cells (Bintig et al., 2012; Weksler et al., 2013; Helms et al., 2016; Bader et al., 2017; Luo et al., 2019) and provides a predictive model to investigate how endothelial Ca^2+^ signals are shaped and regulate the Ca^2+^-dependent production of vasorelaxing mediators, such as nitric oxide (NO), at the human blood-brain barrier (Negri et al., 2021c; Soda et al., 2023). Several studies have shown that neurotransmitters and neuromodulators, such as acetylcholine, glutamate, γ-aminobutyric (GABA), and histamine, evoke NO release from hCMEC/D3 cells through an increase in [Ca^2+^]_i_ that is patterned by InsP_3_-induced ER Ca^2+^ release, NAADP-induces lysosomal Ca^2+^ mobilization, and SOCE (Zuccolo et al., 2019b; Berra-Romani et al., 2020; Negri et al., 2020; Negri et al., 2022). Herein, we demonstrate that the lysosomal TRPML1 is also expressed in hCMEC/D3 cells. We also provide the first evidence that TRPML1-mediated endothelial Ca^2+^ signals are supported by ER Ca^2+^ release through InsP_3_Rs and by SOCE. We further show that the genetic and pharmacological blockade of TRPML1 reduced the ER Ca^2+^ load. Moreover, TRPML1-mediated global Ca^2+^ signals lead to robust NO release that may regulate a variety of processes, including an increase in local cerebral blood flow (CBF), at the neurovascular unit. Finally, we provide the first evidence that TRPML1 support agonist-induced intracellular Ca^2+^ release and NO production in vascular endothelial cells. These findings hint at TRPML1 as an additional component of the endothelial Ca^2+^ toolkit that may be recruited by extracellular autacoids to regulate the endothelial Ca^2+^-dependent functions.

## 2 Materials and methods

### 2.1 Cell culture

Human cerebral microvascular endothelial cells (hCMEC/D3) were obtained from Institut National de la Santé et de la Recherche Médicale (INSERM, Paris, France). hCMEC/D3 cells between passage 25 and 35 were used and cultured as described in (Negri et al., 2022; Berra-Romani et al., 2023). The cells were seeded at a concentration of 27.000 cells/cm^2^ and grown in tissue culture flasks coated with 0.1 mg/mL rat tail Collagen type 1, in the following medium: EBM-2 medium (Lonza, Basel, Switzerland) supplemented with 5% fetal bovine serum, 1% Penicillin-Streptomycin, 1.4 μM hydrocortisone, 5 μg/mL ascorbic acid, 1/100 chemically defined lipid concentrate (Life Technologies, Milan, Italy), 10 mM HEPES and 1 ng/mL basic fibroblast growth factor. The cells were cultured at 37 °C, 5% CO_2_ saturated humidity.

### 2.2 Solutions

Physiological salt solution (PSS) had the following composition (in mM): 150 NaCl, 6 KCl, 1.5 CaCl_2_, 1 MgCl_2_, 10 Glucose, 10 Hepes. In Ca^2+^-free solution (0Ca^2+^), Ca^2+^ was substituted with 2 mM NaCl, and 0.5 mM EGTA was added. Solutions were titrated to pH 7.4 with NaOH. The osmolality of PSS as measured with an osmometer (Wescor 5,500, Logan, UT, United StatesA) was 300–310 mOsm/L.

### 2.3 [Ca^2+^]_i_ and NO imaging

Cells were on glass gelatin-coated coverslips at a density of 5,000 cells/cm^2^ for 24–48 h (Mussano et al., 2020). Cells were next loaded the selective Ca^2+^-fluorophore Fura-2 acetoxymethyl ester (2 µM Fura-2/AM; Thermo Fisher Scientific, Waltham, MA, United States) in PSS for 30 min at 37°C and 5% CO_2_, as described in (Negri et al., 2022; Berra-Romani et al., 2023). After washing in PSS, the coverslip was fixed to the bottom of a Petri dish and the cells were observed by an upright epifluorescence Axiolab microscope (Carl Zeiss, Oberkochen, Germany), usually equipped with a Zeiss ×40 Achroplan objective (water-immersion, 2.0 mm working distance, 0.9 numerical aperture). The cells were excited alternately at 340 and 380 nm, and the emitted light was detected at 510 nm. A first neutral density filter (1 or 0.3 optical density) reduced the overall intensity of the excitation light, and a second neutral density filter (optical density = 0.3) was coupled to the 380 nm filter to approach the intensity of the 340 nm light. A round diaphragm was used to increase the contrast. The excitation filters were mounted on a filter wheel (Lambda 10, Sutter Instrument, Novato, CA, United States). Custom software, working in the LINUX environment, was used to drive the camera (Extended-ISIS Camera, Photonic Science, Millham, United Kingdom) and the filter wheel, and to measure and plot online the fluorescence from 10 up to 40 rectangular “regions of interest” (ROIs). Each ROI was identified by a number. Since cell borders were not clearly identifiable, a ROI may not include the whole cell or may include part of an adjacent cell. Adjacent ROIs never superimposed. [Ca^2+^]_i_ was monitored by measuring, for each ROI, the ratio of the mean fluorescence emitted at 510 nm when exciting alternatively at 340 and 380 nm (F_340_/F_380_). An increase in [Ca^2+^]_i_ causes an increase in the ratio (Negri et al., 2022; Berra-Romani et al., 2023). Ratio measurements were performed and plotted online every 3 s. The experiments were performed at room temperature (22 °C). The Fe^2+^ quench experiments were performed as described in (Kilpatrick et al., 2016) by replacing CaCl_2_ with an equimolar amount of FeCl_2_ and measuring the quenching of Fura-2 fluorescence at 360 nm, i.e., the isosbestic point for Fura-2.

To evaluate NO release, hCMEC/D3 cells were loaded with 4-Amino-5-methylamino-2′,7′-difluorofluorescein diacetate (1 μM, DAF-FM DA) for 60 min in PSS at 22°C, as illustrated in (Negri et al., 2020; Berra-Romani et al., 2023). DAF-FM fluorescence was measured by using the same imaging setup described above for Ca^2+^ measurements but with a different filter set, i.e., excitation at 480 nm and emission at 535 nm wavelength (emission intensity denoted as NO_i_ (F_535_/F_0_)). The changes in DAF-FM fluorescence evoked by extracellular stimulation were recorded and plotted online every 5 s. Measurements of NO were performed at 22 °C. The cellular production of NO was reported as relative fluorescence (F/F_0_) of DAF-FM DA, where F is the fluorescence intensity obtained during recordings and F_0_ is the basal fluorescence intensity.

### 2.4 Immunoblotting

Cells were seeded on a 6-well culture plate, grown to a confluency of 70%–80%, and then silenced or not, according to the protocol described below. For cell lysis, plates were kept on ice and cells were washed twice in ice-cold PBS, scraped with RIPA buffer (Pierce^®^ RIPA Buffer, Thermo Fisher Scientific, Waltham, MA, United States) and protease inhibitor cocktail (Halt™ Protease Inhibitor Cocktail, 1:100, Thermo Fisher Scientific, Waltham, MA, United States). Lysates were vortexed and kept on ice for 10 min, then centrifuged at 4 °C for 15 min at 13,000× *g*. Protein concentrations were determined by using a Bicinchoninic Acid (BCA) kit (Merck KGaA, Darmstadt, Germany) following the manufacturer’s instructions. 20 μg of lysates were resuspended in SDS loading buffer, heated 30 min at 37 C and then separated on 4%–15% Mini-PROTEAN TGX Precast Protein Gels Bio-Rad (Bio-Rad, Hercules, CA; United States). Then, the proteins were transferred out of the gel on to the PVDF Membrane (Trans-Blot Turbo Transfer Pack, Bio-Rad, Hercules, CA; United States) with the Trans-Blot Turbo Transfer apparatus (BioRad, Hercules, CA; United States). Membranes were blocked by incubation for 1 h at room temperature in TBST (20 mM Tris, 150 mM NaCl, 0.1% Tween 20, pH 7.6) 5% BSA solution and then incubated in agitation overnight at 4 °C with rabbit anti–TRPML1 (#ACC-081, 1:200 in TBST 5% BSA 0.02% sodium azide; Alomone, Jerusalem, Israel) and anti-β-Actin-Peroxidase (#A385416, 1:1,000 in TBST 5% BSA 0.02% sodium azide; Merck KGaA, Darmstadt, Germany) antibodies. Membranes were then washed with TBST and incubated with the appropriate HPR–conjugated antibody (anti-rabbit HRP #31460, 1:2,000 in TBST 5% BSA; Thermo Fisher Scientific, Waltham, MA, United States). Differences in protein expression was evaluated by using Fiji (ImageJ software).

### 2.5 Immunofluorescence

Cells were cultured on Collagen Type I (#C3867, Merck KGaA, Darmstadt, Germany) coated glass coverslips and used after 80%–90% confluency. Cells were washed and then incubated with LysoTracker™ Red DND-99 (#L7528, 50 nM; Thermo Fisher Scientific, Waltham, MA, United States) for 1 h. After incubation, cells were washed twice with Hanks’ Balanced Salt Solution (HBSS), fixed in 4% paraformaldehyde at room temperature for 10 min and permeabilized with 0.1% Triton X-100 in PBS for 10 min. Cells were then blocked with 1% BSA in PBS and incubated overnight at 4 °C with the following primary antibodies: anti-TRPML1 (#ACC-081; Alomone Labs, Jerusalem, Israel) 1:200 in TBST 1% BSA; anti-LAMP1 (#ab25630, Abcam, Cambridge, United Kingdom) 1:100 in TBST 5% BSA. Bound antibodies were detected with Alexa Fluor secondary antibodies (#A11008, #A21235, 1:500, Alexa Fluor^**TM**^ 488; Thermo Fisher Scientific, Waltham, MA, United States). Images were collected on a Leica SP8 Confocal scanning microscope using oil immersion 60x (HC PL APO 63x/1.40 OIL CS2 UV, Leica, Wetzlar, Germany) objectives.

### 2.6 Gene silencing

Genetic deletion of *MCOLN1*, which encodes for TRPML1, was carried by using a similar approach to that described in (Mussano et al., 2020; Negri et al., 2020). Cells were transiently transfected with the esiRNA targeting TRPML1 (EHU062561, MISSION^®^ esiRNA, 100 nM final concentration) purchased from Merck (Merck KGaA, Darmstadt, Germany) by using the Lipofectamine™ RNAiMAX Transfection Reagent (Thermo Fisher Scientific, Waltham, MA, United States) protocol in Opti-MEM™ I Reduced Serum Medium (Thermo Fisher Scientific, Waltham, MA, United States), according to manufacturer’s instructions. 4 h after transfection, the esiRNA-Lipofectamin complex was eliminated and fresh culture media containing 5% FBS was added to the cells. Cells were then kept in incubator at 37°C and 5% CO_2_ and allowed to grow according to the protocol to be used. The effectiveness of silencing was determined by immunoblotting and the silenced hCMEC/D3 cells were used 48 h after transfection. The Trypan blue exclusion assay confirmed that the genetic silencing of TRPML1 did not affect hCMEC/D3 cell viability (Supplementary Figure S1).

### 2.7 Statistics

All the data have been obtained from at least three independent experiments on hCMEC/D3 cells. The peak amplitude of agonist-evoked Ca^2+^ signals was measured as the difference between the ratio (F_340_/F_380_) at the Ca^2+^ peak and the mean ratio of 30 s baseline before the peak.

Data were analyzed with GraphPad Prism 7 (GraphPad Software, Inc., La Jolla, CA, United States). Preliminary Shapiro-Wilk test was performed to check the normal distributions of each dataset: accordingly, statistical analysis was performed by using either the non-parametric tests (Mann-Whitney test or Kruskal–Wallis test) or parametric tests (Student’s t-test or one-way ANOVA test). A *p*-value of <0.05 was considered significant.

### 2.8 Chemicals

ML-SAI (# SML0627), ML-SI3 (GW405833 hydrochloride; # G1421), CPA (Ciclo piazonic acid; # C1530), 2-APB (# D9754), BTP-2 (# 203890 CRAC channel inhibitor BTP2), Pyr6 (# SML1241), were purchased from Merck GKaA (Darmstadt, Germany). BAPTA/AM (#196419) and L-NIO (L-N⁵-1-Iminoethyl ornithine, Dihydrochloride; # 400600) were purchased from Merck Millipore (Burlington, Massachusetts, United States). Nigericin sodium salt (# 4,312) was purchased from Tocris Bioscience (Bristol, United Kingdom). PI(3,5)P_2_ diC8) (#P-3508) was purchased from Echelon Biosciences Inc. (Salt Lake City, United States). All other chemicals were of analytical grade and purchased from Sigma Chemical Co. (Milan, Italy).

## 3 Results

### 3.1 Lysosomal TRPML1 is expressed in hCMEC/D3 cells

**FIGURE 1:**
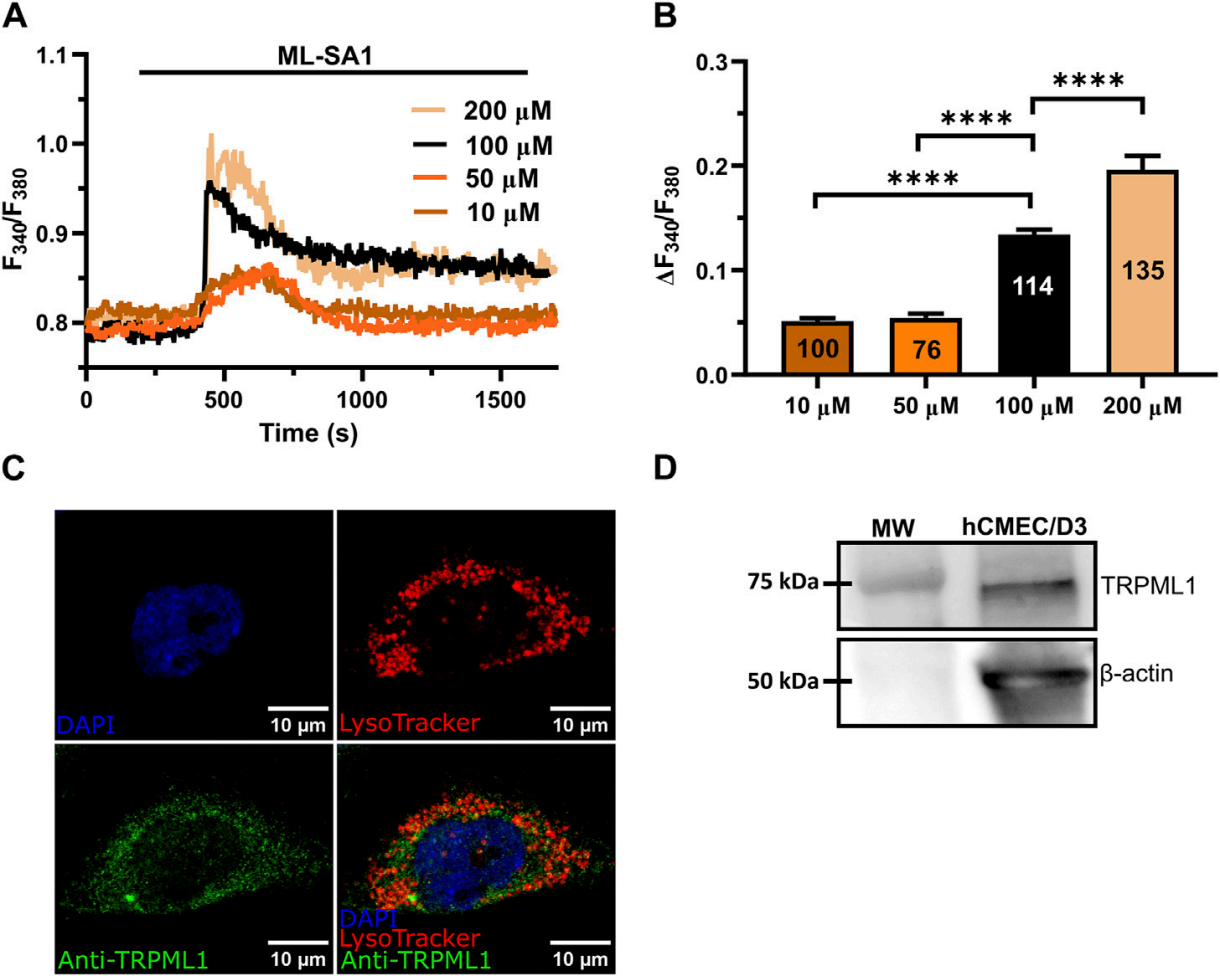
ML-SA1 evokes a dose-dependent increase in [Ca^2+^]_i_ in hCMEC/D3 cells. **(A)** Intracellular Ca^2+^ signals evoked by increasing concentration of ML-SA1 in hCMEC/D3 cells. **(B)** Mean ± SEM of the peak amplitude of ML-SA1-induced Ca^2+^ responses in hCMEC/D3 cells at different agonist concentrations. **** indicates *p* < 0.0001 (Student’s t-test and Mann-Whitney test). **(C)** Confocal fluorescence images of hCMEC/D3 cells loaded with LysoTracker-Red DND-99 (red) to mark acidic organelles and stained with an antibody against TRPML1 antibody (green). Nuclei were stained using DAPI (blue). Scale bar: 10 µm. **(D)** Representative western blotting analysis of TRPML1 on hCMEC/D3 cells lysates. Major bands of the expected molecular weights (MW) for TRPML1 (75 kDa) and the loading control protein β-actin (50 kDa) is indicated.

The synthetic agonist, ML-SAI, has been widely used to evaluate the functional expression of functional TRPML1 channels in a variety of cell lines (Kilpatrick et al., 2016; Thakore et al., 2020; Tedeschi et al., 2021; Boretto et al., 2023; Scorza et al., 2023). In hCMEC/D3 cells loaded with the ratiometric Ca^2+^-sensitive fluorophore, Fura-2, ML-SAI induced a dose-dependent increase in [Ca^2+^]_i_ that was evident at concentrations ≥10 µM (Figure 1A). At 10–50 μM, ML-SA1 caused a slow but transient increase in [Ca^2+^]_i_ that started with some delay after the application of the agonist (Figure 1A). Higher concentrations of ML-SA1 (100 μM and 200 µM) induced a biphasic Ca^2+^ signal following the slow increase in [Ca^2+^]_i_ and representing a rapid Ca^2+^ peak followed by a sustained plateau level (Figure 1A). The amplitude of the Ca^2+^ response to increasing concentrations of ML-SA1 has been reported in Figure 1B. Immunofluorescence showed that punctate vesicular structures could be detected in hCMEC/D3 cells stained with the lysosomal marker LAMP-1 (Supplementary Figure S2A) and Lysotracker Red (Supplementary Figure S2B), a red fluorescent weak base that is selective for acidic organelles (Faris et al., 2019; Scorza et al., 2023). Consistent with this, nigericin (50 µM), a H^+^/K^+^ ionophore that alkalinizes the lysosomal pH (Morgan et al., 2011; Riva et al., 2018; Morgan and Galione, 2021), erased Lysotracker Red fluorescence (Supplementary Figure S2C), confirming that it selectively labels lysosomal vesicles. Co-immunofluorescence analysis showed that both Lysotracker Red and a TRPML1 specific antibody exhibited a similar punctate distribution throughout the cells (Figure 1C). Finally, immunoblotting with a TRPML1-specific antibody detected a major band of ∼75 kDa (Figure 1D), which is the predictive molecular weight for the TRPML1 protein (Tedeschi et al., 2021). Taken together, these findings show that the lysosomal TRPML1 is expressed and triggers global Ca^2+^ signals in hCMEC/D3 cells.

### 3.2 TRPML1-mediated global Ca^2+^ signals are sustained by lysosomal Ca^2+^ release and extracellular Ca^2+^ entry

**FIGURE 2:**
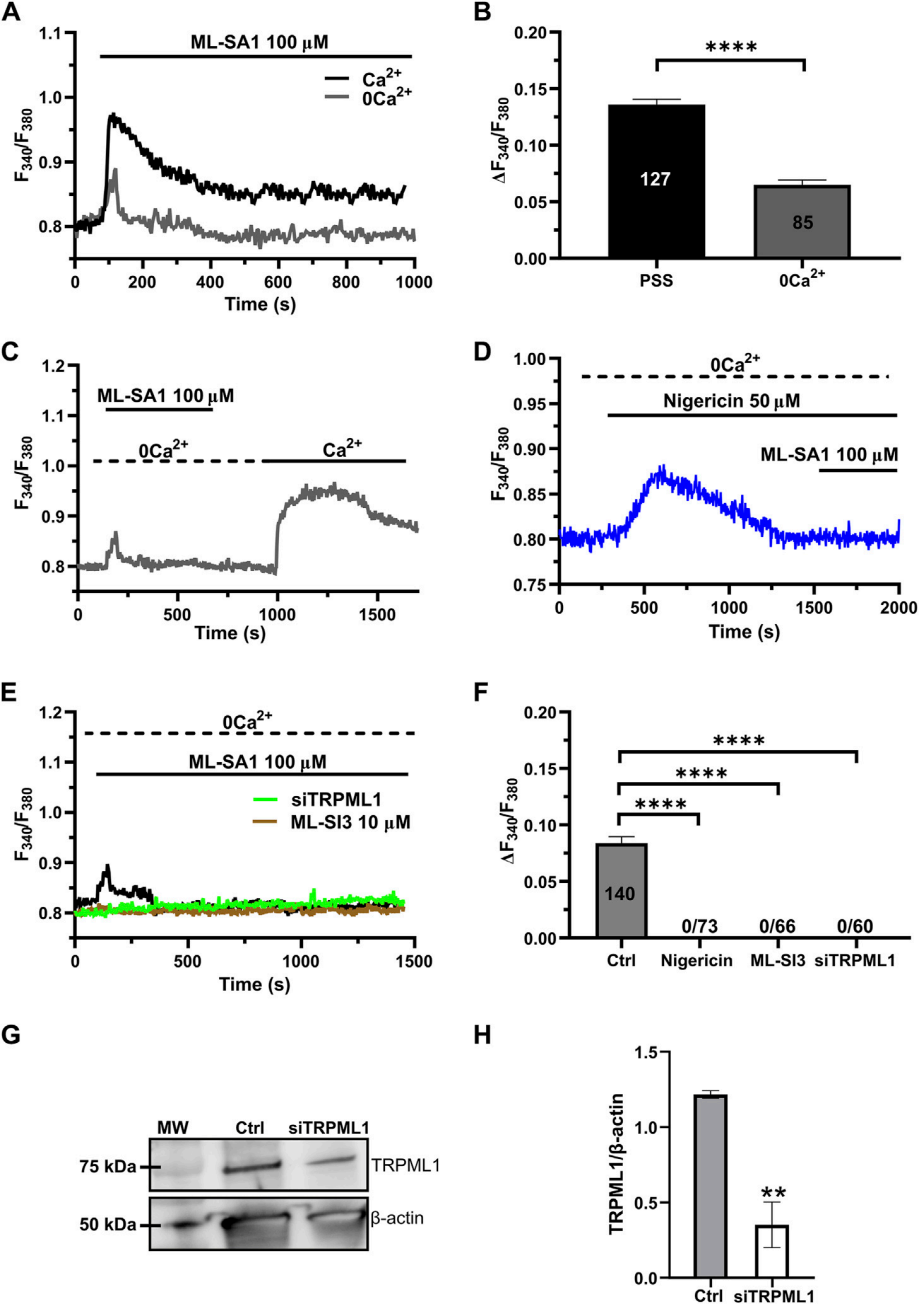
Lysosomal Ca^2+^ release and extracellular Ca^2+^ entry sustain TRPML-mediated Ca^2+^ signals hCMEC/D3 cells. **(A)** ML-SA1 induced a small transient increase in [Ca^2+^]_i_ in the absence of extracellular Ca^2+^. **(B)** Mean ± SEM of the peak amplitude of the TRPML1-induced Ca^2+^ responses in presence and absence of extracellular Ca^2+^. **** indicates *p <* 0.0001 (Student’s t-test). **(C)** Restoration of extracellular Ca^2+^ upon removal of the agonist resulted in a second bump in [Ca^2+^]_i_, which was indicative of SOCE. **(D)** Pre-incubation with nigericin (50 μM, 20 min) under 0Ca^2+^ conditions, induced a transient increase in [Ca^2+^]_i_, reflecting the depletion of the Ca^2+^ store. The subsequent application of ML-SA1 failed to induce a significant Ca^2+^ response in hCMEC/D3 cells. **(E)** Pharmacological (ML-SI3; 10 μM, 20 min) and genetic (siTRPML1) inhibition of TRPML1, totally abolished the Ca^2+^ response under 0Ca^2+^ condition. **(F)** Mean ± SEM of the amplitude of Ca^2+^ responses in cells under the designated treatments. **** indicates *p <* 0.0001 (Kruskal–Wallis one-way Anova test followed by Dunn’s *post hoc* test). **(G)** Representative western blotting analysis of the TRPML1 protein expressed in hCMEC/D3 (Ctrl) and hCMEC/D3 transfected with the specific siTRPML1 (siTRPML1). Major bands of the expected molecular weights (MW) for TRPML1 (75 kDa) and the loading control protein β-actin (50 kDa) is indicated. **(H)** Mean ± SEM of the TRPML1/β-actin ratio expression of four independent western blotting experiments. ** indicates *p <* 0.005 (Student’s t-test).

Recent studies have shown that the global Ca^2+^ response to TRPML1 activation involves both lysosomal Ca^2+^ release and extracellular Ca^2+^ entry across the plasma membrane (Kilpatrick et al., 2016; Tedeschi et al., 2021). The long-lasting plateau phase of the Ca^2+^ responses illustrated in Figure 1A suggests that ML-SA1 at concentrations higher than 50 μM may also activate a Ca^2+^ entry pathway in hCMEC/D3 cells. Consistent with this hypothesis, in the absence of extracellular Ca^2+^ (0Ca^2+^), 100 μM ML-SA1 induced a transient increase in [Ca^2+^]_i_ whose amplitude was significantly (*p* < 0.05) smaller as compared to the amplitude of the Ca^2+^ response recorded in the presence of extracellular Ca^2+^ (Figures 2A,B). Restoration of extracellular Ca^2+^ 300 s after the removal of ML-SA1 induced a prompt increase in [Ca^2+^]_i_ that was obviously independent of the presence of the agonist in the bath (Figure 2C). As discussed in (Morgan et al., 2011; Garrity et al., 2016; Faris et al., 2019; Lloyd-Evans and Waller-Evans, 2019; Moccia et al., 2021b), depletion of the lysosomal Ca^2+^ pool with nigericin has long been used as a pharmacological approach to confirm that ML-SA1 and NAADP mobilize the lysosomal Ca^2+^ store. Consistent with this, the application of nigericin (50 µM) under 0Ca^2+^ conditions induced a transient increase in [Ca^2+^]_i_ reflecting the depletion of the lysosomal Ca^2+^ content (Figure 2D). The subsequent application of 100 μM ML-SA1 failed to induce a discernible Ca^2+^ response in hCMEC/D3 cells (Figures 2D,F). In addition, ML-SA1-induced intracellular Ca^2+^ release was abolished by ML-SI3 (10 µM) (Figures 2E,F), a specific TRPML1 antagonist (Wang et al., 2015; Kilpatrick et al., 2016; Boretto et al., 2023), and by the genetic deletion of TRPML1 with a selective small interfering RNA (siTRPML1) (Figures 2E,F). The efficacy of the siTRPML1-mediated reduction in TRPML1 protein expression has been illustrated in Figures 2G,H. Taken together, these findings show that TRPML1-mediated global Ca^2+^ signals are triggered by lysosomal Ca^2+^ release and sustained by extracellular Ca^2+^ entry.

### 3.3 ER Ca^2+^ release through InsP_3_Rs and SOCE contribute to TRPML1-mediated global Ca^2+^ signals in hCMEC/D3 cells

**FIGURE 3:**
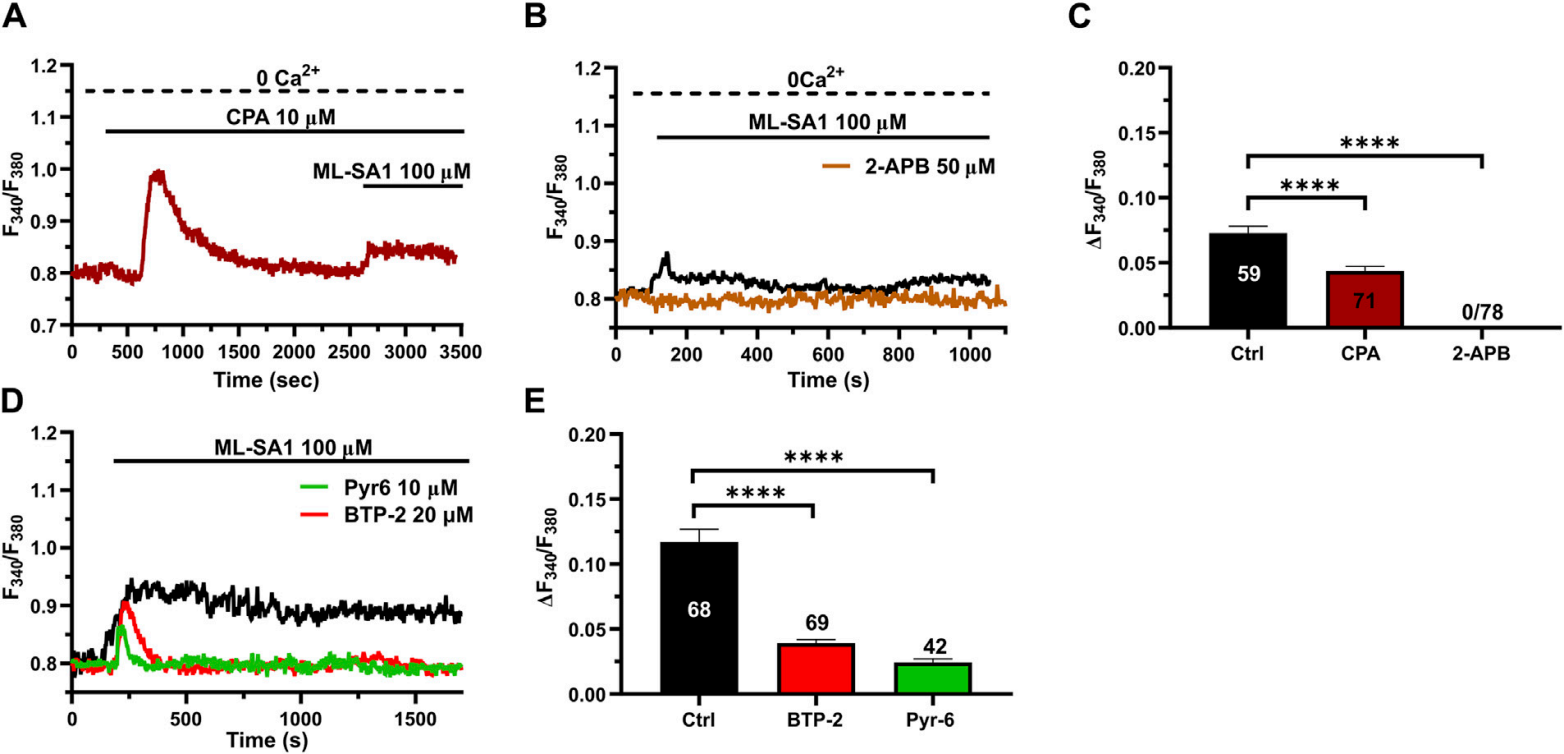
ER Ca^2+^ release and SOCE contribute to the TRPML1-mediated Ca^2+^ signals in hCMEC/D3 cells. **(A, B)** pre-incubation with CPA (10 μM, 30 min) or 2-APB (50 μM, 30 min) significantly reduced the ML-SA1-induced Ca^2+^ response under 0Ca^2+^ conditions. **(C)** Mean ± SEM of the amplitude of the Ca^2+^ responses in cells under the designated treatments. **** indicates *p <* 0.0001 (Kruskal–Wallis one-way Anova test followed by Dunn’s *post hoc* test). **(D)** The Orai1 blockers, BTP-2 (20 μM, 20 min) and Pyr6 (20 μM, 20 min), reduced the ML-SA1-evoked Ca^2+^ response. **(E)** Mean ± SEM of the amplitude of Ca^2+^ responses in cells under the designated treatments. Each drug totally inhibited the Ca^2+^ response. **** indicates *p <* 0.0001 (Kruskal–Wallis one-way Anova test followed by the Dunn’s *post hoc* test).

The ER is the major Ca^2+^ store that may amplify spatially restricted lysosomal Ca^2+^ microdomains into global increases in [Ca^2+^]_i_ (Morgan, 2016; Moccia et al., 2023c; Galione et al., 2023). Furthermore, the evidence mentioned previously that ML-SA1-induced extracellular Ca^2+^ entry may occur even in the absence of the agonist of the bath is reminiscent of SOCE activation (Murata et al., 2007; Bird et al., 2008; Moccia et al., 2023a), further confirming the contribution of the ER Ca^2+^ store between the initial event of lysosomal Ca^2+^ release and the subsequent influx of Ca^2+^. Consistent with this, the intracellular Ca^2+^ response to 100 μM ML-SA1 was significantly (*p* < 0.05) reduced by the prior incubation with cyclopiazonic acid (CPA; 20 µM) under 0Ca^2+^ conditions to deplete the ER Ca^2+^ store (Figure 3A) and by 2-aminoethoxy diphenyl borate (2-APB; 50 µM) (Figure 3B), a reliable InsP_3_R blocker in the absence of extracellular Ca^2+^ (Pafumi et al., 2015). The inhibitory effect of CPA and 2-APB on ML-SA1-induced intracellular Ca^2+^ release has been illustrated in Figure 3C hCMEC/D3 cells do not express RyRs (Zuccolo et al., 2019b) and, therefore, we did not assess their contribution to ML-SA1-induced intracellular Ca^2+^ release. SOCE is the main pathway that sustains Ca^2+^ entry during ER Ca^2+^ release events through InsP_3_Rs in vascular endothelial cells (Blatter, 2017; Groschner et al., 2017; Moccia et al., 2023a), including hCMEC/D3 cells (Berra-Romani et al., 2020; Negri et al., 2020; Negri et al., 2021a; Negri et al., 2022). Consistent with this, 100 μM ML-SA1 induced a transient and tiny Ca^2+^ response in the presence of 20 µM BTP-2 (Figures 3D,E) or 10 µM Pyr6 (Figures 3D,E), which are two established blockers of Orai1 (Schleifer et al., 2012; Chauvet et al., 2016; Zuccolo et al., 2016; Zhang et al., 2020), which serves as store-operated Ca^2+^ channel in hCMEC/D3 cells (Zuccolo et al., 2019b; Negri et al., 2020). However, TRPML1 can directly mediate Ca^2+^ entry across the plasma membrane (Kilpatrick et al., 2016). TRPML1 is also permeable to Fe^2+^, which causes a significant quench in Fura-2 fluorescence when it enters the cytosol (Kilpatrick et al., 2016). Therefore, hCMEC/D3 cells were challenged with FeCl_2_ (1 mM) under 0Ca^2+^ conditions after stimulation with ML-SA1 (100 μM), as shown in (Kilpatrick et al., 2016). Supplementary Figure S3 shows that FeCl_2_ did not induce any detectable quench in Fura-2 fluorescence, thereby suggesting TRPML1 does not directly contribute to ML-SA1-evoked Ca^2+^ entry. Taken together, these findings demonstrate that TRPML1-mediated global Ca^2+^ signals in hCMEC/D3 cells are supported by ER Ca^2+^ release through InsP_3_Rs and by SOCE activation.

### 3.4 TRPML1 regulates ER Ca^2+^ load in hCMEC/D3 cells

**FIGURE 4:**
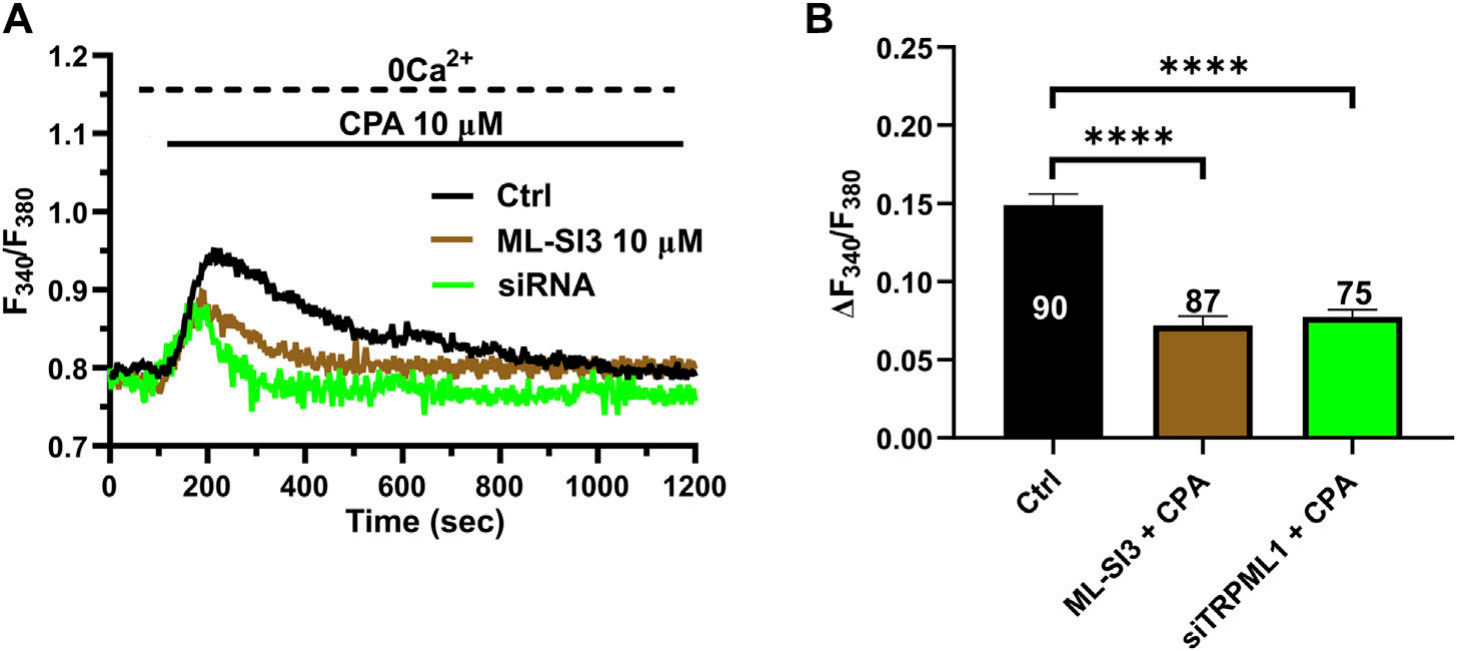
The inhibition of TRPML1 reduces ER Ca^2+^ release. **(A)** The transient Ca^2+^ response induced by CPA (10 µM) under 0 Ca^2+^ conditions is strongly reduced in the presence of ML-SI3 (10 μM, 20 min) and in hCMEC/D3 cells transfected with the selective siTRPML1. **(B)** Mean ± SEM of the amplitude of the Ca^2+^ responses in cells under the designated treatments. **** indicates *p <* 0.0001 (Kruskal–Wallis one-way Anova test followed by the Dunn’s *post hoc* test).

**FIGURE 5:**
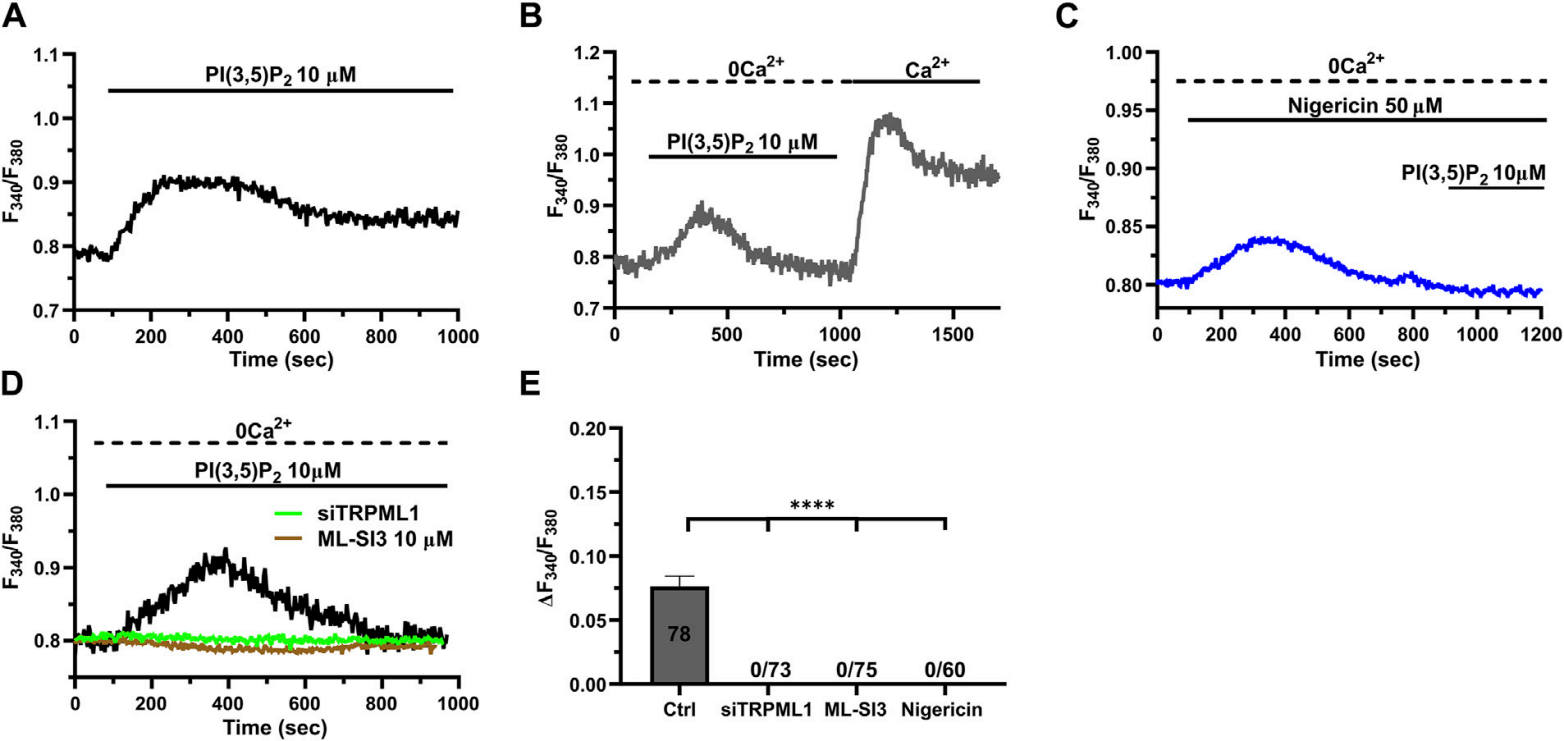
The endogenous ligand PI(3,5)P_2_ mimics the Ca^2+^ response to ML-SA1. **(A)** 10 µM PI(3,5)P_2_ induced a long-lasting increase in [Ca^2+^]_i_, mimicking the ML-SA1-evoked Ca^2+^ response. **(B)** Restoration of extracellular Ca^2+^ upon removal of the agonist PI(3,5)P_2_ resulted in a second bump in [Ca^2+^]_i_, which was indicative of SOCE. **(C)** Depletion of lysosomal Ca^2+^ stores with 50 µM nigericin abolished the PI(3,5)P_2_ –induced Ca^2+^ response. **(D)**. Pharmacological (ML-SI3; 10 μM, 20 min) and genetic (siTRPML1) inhibition of TRPML1 totally abolished the PI(3,5)P_2_ –induced Ca^2+^ response, in absence and presence of extracellular Ca^2+^. **(E)** Mean ± SEM of the amplitude of Ca^2+^ responses in the absence of extracellular Ca^2+^ (0Ca^2+^) under the designated treatments. Each drug totally inhibited the Ca^2+^ response. **** indicates *p <* 0.0001 (Kruskal–Wallis one-way Anova test followed by the Dunn’s *post hoc* test).

TRPML1 could promote ER Ca^2+^ release by directly activating the juxtaposed InsP_3_Rs via Ca^2+^-induced Ca^2+^ release (CICR) (Thakore et al., 2020) or by loading the ER with Ca^2+^ in a SERCA-dependent manner, thereby activating InsP_3_Rs via the ER Ca^2+^ overload (Macgregor et al., 2007; PMID: 17387177). A recent study showed that TRPML1 regulates the ER Ca^2+^ content in primary rat cortical neurons (Tedeschi et al., 2021). Therefore, we reasoned that, if TRPML1 did the same in hCMEC/D3 cells, CPA-evoked ER Ca^2+^ release would be impaired by interfering with TRPML1-mediated Ca^2+^ mobilization. In agreement with this hypothesis, the ER Ca^2+^ response to CPA (20 μM) was significantly (*p* < 0.05) reduced by blocking TRPML1 with either ML-SI3 (10 µM) (Figure 5A) or the siTRPML1 (Figure 4A). The statistical analysis of these experiments has been illustrated in Figure 4B. Collectively, these findings strongly suggests that TRPML1-mediated lysosomal Ca^2+^ release leads to an increase in ER Ca^2+^ content, which is decreased upon its inhibition. Therefore, we conclude that TRPML1 may mediate ER Ca^2+^ content and lead to InsP_3_ activation upon an increase in luminal Ca^2+^ (Missiaen et al., 1992).

### 3.5 TRPML1 mediates PI(3,5)P_2_-evoked Ca^2+^ signals in hCMEC/D3 cells

The lysosomal phosphoinositide PI(3,5)P_2_ is regarded as a putative endogenous ligand of TRPML1 (Dong et al., 2010; Zhang et al., 2012; Isobe et al., 2019; Li et al., 2019; Negri et al., 2021b). In order to assess whether PI(3,5)P_2_ was also able to induce TRPML1-mediated global Ca^2+^ signals in hCMEC/D3 cells, we challenged the cells with PI(3,5)P_2_ diC8, a cell-permeable analog of PI(3,5)P_2_ (Tedeschi et al., 2021). 10 μM PI(3,5)P_2_ diC8 induced a slowly rising, but long-lasting increase in [Ca^2+^]_i_ (Figure 5A). The Ca^2+^ add-back protocol confirmed that, under 0Ca^2+^ conditions, 10 µM PI(3,5)P_2_ diC8 induced a transient increase in [Ca^2+^]_i_ that was followed by a second elevation in [Ca^2+^]_i_ upon restoring extracellular Ca^2+^ in the absence of the agonist (Figure 5B). As described above for ML-SA1, previous depletion of the lysosomal Ca^2+^ store with nigericin (50 µM) abolished PI(3,5)P_2_ diC8-induced intracellular Ca^2+^ release in the absence of extracellular Ca^2+^ (0Ca^2+^) (Figure 5C). Furthermore, the intracellular Ca^2+^ response to PI(3,5)P_2_ diC8 was significantly (*p* < 0.05) reduced by both ML-SI3 (10 μM; Figure 5D) and by the selective siTRPML1 (Figure 5D). The inhibitory effect of nigericin, ML-SI3, and siTRPML1 on PI(3,5)P_2_ diC8-induced intracellular Ca^2+^ release has been illustrated in Figure 5E. Collectively, these findings provide the evidence that PI(3,5P)_2_ may also serve as an endogenous agonist of TRPML1 in hCMEC/D3 cells.

### 3.6 TRPML1-mediated global Ca^2+^ signals lead to NO release in hCMEC/D3 cells

**FIGURE 6:**
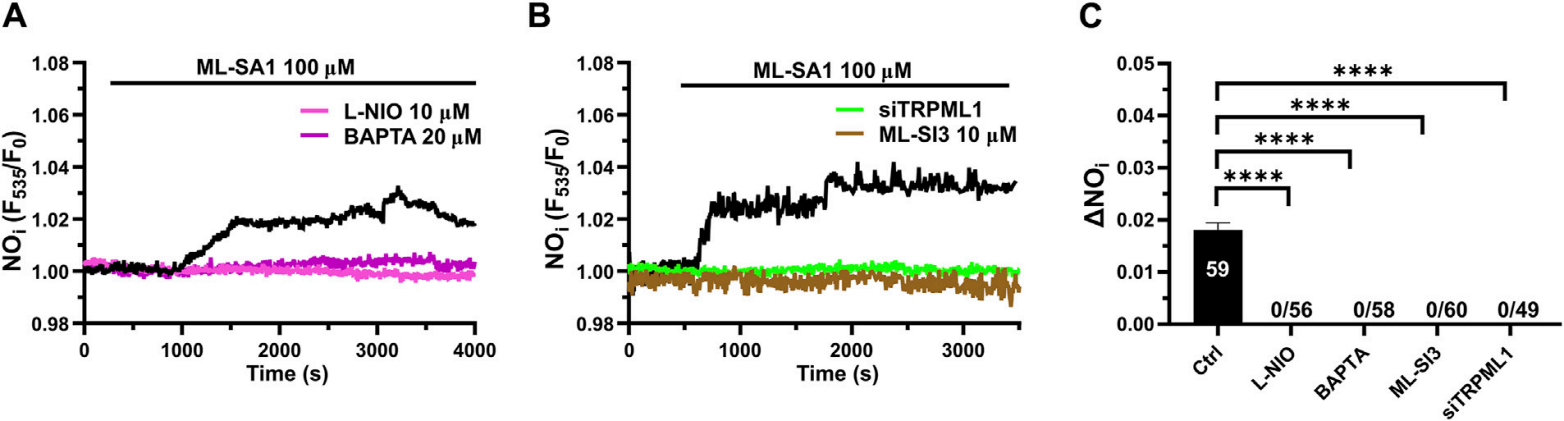
TRPML1 induces Ca^2+^-dependent NO release in hCMEC/D3 cells. **(A)** 100 μM ML-SA1 evoked a slow, but sustained increase in DAF-FM fluorescence, which reflected NO release and was inhibited by L-NIO (10 μM, 1 h) and BAPTA-AM (20 μM, 2 h). **(B)** Pharmacological (ML-SI3; 10 μM, 20 min) and genetic (siTRPML1) inhibition of TRPML1, significantly abolished the ML-SA1-induced NO response. **(C)** Mean ± SEM of the amplitude of NO responses in presence of extracellular Ca^2+^. **** indicates *p <* 0.0001 (Kruskal–Wallis one-way Anova test followed by the Dunn’s *post hoc* test).

Recent studies have shown that a global increase in [Ca^2+^]_i_ can result in robust NO release from hCMEC/D3 cells (Berra-Romani et al., 2020; Negri et al., 2020; Negri et al., 2022; Berra-Romani et al., 2023). In order to assess whether TRPML1-mediated global Ca^2+^ signals could also induce NO production, we loaded hCMEC/D3 cells with the NO-sensitive fluorophore, DAF-FM DA. We found that 100 μM ML-SA1 induced a slow, but sustained increase in DAF-FM fluorescence (Figure 6A), which was indicative of NO release (Sheng et al., 2005; Sheng and Braun, 2007; Greenberg et al., 2017; Berra-Romani et al., 2020; Negri et al., 2020; Negri et al., 2022; Berra-Romani et al., 2023). ML-SAI-induced NO release was significantly inhibited by BAPTA-AM (20 μM; Figure 6A), a membrane permeable buffer of intracellular Ca^2+^ levels, and by L-NIO (50 μM; Figure 6A), a specific eNOS inhibitor. The inhibitory effect of BAPTA and L-NIO on ML-SA1-induced NO release has been illustrated in Figure 5C. Moreover, ML-SI3 (10 µM) and siTRPML1 significantly (*p* < 0.05) inhibited ML-SA1-induced NO release (Figures 6B,C). Therefore, the lysosomal TRPML1 is able to trigger massive NO production in the human cerebrovascular endothelial cell line, hCMEC/D3.

### 3.7 TRPML1 supports ATP-induced Ca^2+^ signaling and NO release in hCMEC/D3 cells

**FIGURE 7:**
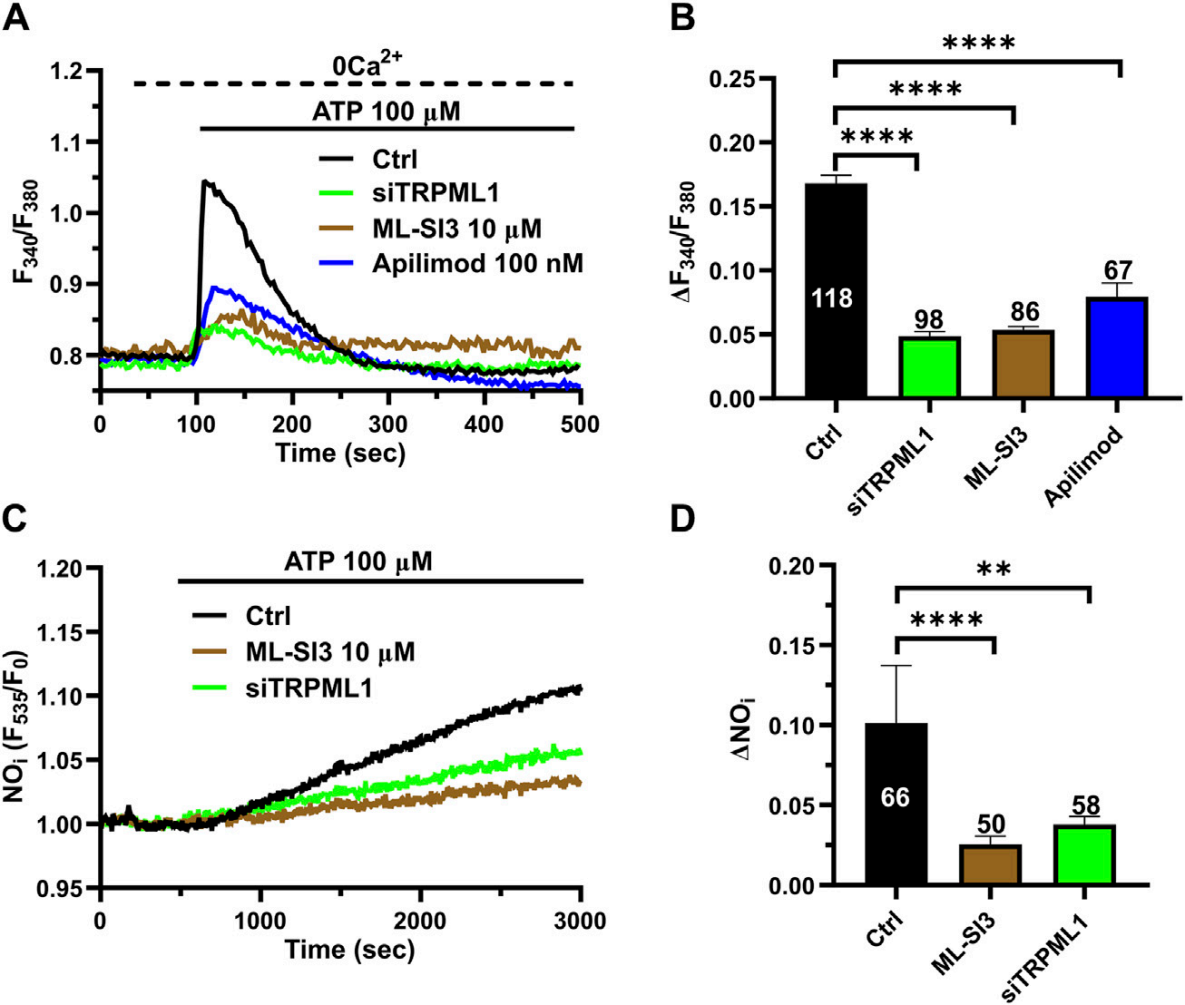
TRPML1 mediates ATP-induced intracellular Ca^2+^ release and NO production. **(A)** The Ca^2+^ response to ATP (100 μM) under 0 Ca^2+^ conditions was strongly reduced in the presence of ML-SI3 (10 μM, 20 min) or apilimod (100 nM, 20 min) and in hCMEC/D3 cells transfected with the selective siTRPML1. **(B)** Mean ± SEM of the amplitude of the Ca^2+^ responses in cells under the designated treatments. **** indicates *p <* 0.0001 (Kruskal–Wallis one-way Anova test followed by Dunn’s *post hoc* test). **(C)**. ATP (100 μM) induced a sustained increase in DAF-DM fluorescence, which reflected NO release and was inhibited in the presence of ML-SI3 (10 μM, 20 min) and in hCMEC/D3 cells transfected with the selective siTRPML1. **(D)** Mean ± SEM of the amplitude of the Ca^2+^ responses in cells under the designated treatments. **** indicates *p <* 0.0001 (Kruskal–Wallis one-way Anova test followed by the Dunn’s *post hoc* test).

The evidence that lysosomal Ca^2+^ release through TRPML1 controls ER Ca^2+^ release and induce InsP_3_R-mediated Ca^2+^ signaling strongly suggests that TRPML1 may support agonist-induced Ca^2+^ signals and NO release in hCMEC/D3 cells. ATP has long been the most widely exploited agonist to stimulate endothelial Ca^2+^ signals along the vascular tree (Scarpellino et al., 2019; Scarpellino et al., 2022; Lisec et al., 2024). ATP activates the Gq-coupled P2Y_2_ receptors to elicit InsP_3_-induced ER Ca^2+^ release in hCMEC/D3 cells (Bintig et al., 2012; Forcaia et al., 2021). A recent study showed that ATP-induced Ca^2+^ signaling is primarily mediated by intracellular Ca^2+^ release in hCMEC/D3 cells (Bintig et al., 2012). Herein, we found that ATP (100 μM) evoked a transient increase in [Ca^2+^]_i_ that was significantly (*p* < 0.05) reduced by ML-SI3 (10 µM) (Figure 7A) and siTRPML1 (Figure 7A). We then assessed whether the pharmacological blockade of PYKfive, which is part of the lipid kinase complex that synthetizes PI(3,5)P_2_, with the selective inhibitor, apilimod (Somogyi et al., 2023), affected ATP-induced ER Ca^2+^ release. Apilimod (100 nM) significantly (*p* < 0.05) reduced ATP-induced ER Ca^2+^ release in hCMEC/D3 cells (Figure 7A), while it did not activate *per se* any increase in [Ca^2+^]_i_, as reported in (Hou et al., 2023). The inhibitory effect of ML-SI3, siTRPML1, and apilimod on the intracellular Ca^2+^ response to ATP has been illustrated in Figure 7B. Furthermore, both the pharmacological and genetic blockade of TRPML1-mediated lysosomal Ca^2+^ release with, respectively, ML-SI3 (10 µM) (Figures 7C,D) and the siTRPML1 (Figures 7C,D), significantly reduced ATP-induced NO release in hCMEC/D3 cells loaded with DAF-DM. Overall, these findings indicate that TRPML1, either indirectly (by refilling the ER Ca^2+^ pool) or directly (by activating the ER-embedded InsP_3_Rs via the luminal Ca^2+^ overload) can support agonist-induced endothelial Ca^2+^ signaling at the neurovascular unit.

## 4 Discussion

In the present investigation, we showed that the lysosomal TRPML1 can trigger global Ca^2+^ signals, involving both Ca^2+^ release and Ca^2+^ entry, in human cerebrovascular endothelial cells, as recently shown for HeLa cells and primary cultured human skin fibroblasts (Kilpatrick et al., 2016), rat primary cortical neurons (Tedeschi et al., 2021), and MDA-MB-231 breast cancer cells (Boretto et al., 2023). We further showed that TRPML1-mediated global Ca^2+^ signals lead to robust NO production, which is not only a recognized proxy for endothelial Ca^2+^ signaling, but also a critical vasorelaxing pathway in brain microcirculation. Therefore, the endothelial TRPML1 channel stands out as a novel component of the Ca^2+^ toolkit at the neurovascular unit that could be involved in the regulation of CBF during neuronal activity (Negri et al., 2021c; Longden et al., 2021; Moccia et al., 2022; Mughal et al., 2024). In agreement with this hypothesis, we showed that TRPML1 supports ATP-induced intracellular Ca^2+^ release and NO production in hCMEC/D3 cells.

Lysosomal Ca^2+^ signaling is emerging as an additional regulator of endothelial Ca^2+^ dynamics (Moccia et al., 2021a; Negri et al., 2021b). The lysosomal agonist, nicotinic acid adenine dinucleotide phosphate (NAADP), has been shown to gate endothelial TPCs to regulate mean arterial pressure via NO release (Brailoiu et al., 2010), to stimulate angiogenesis (Favia et al., 2014), von Willebrand factor release (Esposito et al., 2011), and vasculogenesis (Di Nezza et al., 2017; Moccia et al., 2021b). In addition, NAADP-induced lysosomal Ca^2+^ release may interact with InsP_3_-induced ER Ca^2+^ mobilization to shape the Ca^2+^ signal and thereby increase eNOS activity in the human cerebrovascular endothelial cell line, hCMEC/D3, employed in the present investigation (Berra-Romani et al., 2020; Negri et al., 2020; Negri et al., 2021a; Negri et al., 2022). The endothelial role of TRPML1 is less known. A recent study has shown that TRPML1 can increase the interaction between lysosomes and multivesicular bodies in mouse coronary artery endothelial cells, which results in reduced exosome release (Li et al., 2022). The application of low concentrations of ML-SA1 (10 μM), a TRPML1 synthetic agonist, induced a spatially-restricted lysosomal Ca^2+^ signal, as revealed by a GCaMP3-ML1 construct that was designed by expressing the genetic Ca^2+^-indicator, GCaMP3, on the cytoplasmic NH_2_-tail of TRPML1 (Li et al., 2022). Herein, we assessed whether TRPML1 was expressed and able to induce global Ca^2+^ signals in the human cerebrovascular endothelial cell line, hCMEC/D3, as cytosolic Ca^2+^ signals are believed to play a crucial role in endothelium-dependent NO release and CBF regulation at the neurovascular unit (Negri et al., 2021c; Kuppusamy et al., 2021; Longden et al., 2021; Thakore et al., 2021; Moccia et al., 2022; Peters et al., 2022; Mughal et al., 2024).

ML-SA1 induced a dose-dependent global Ca^2+^ signal consisting of a slow rise in [Ca^2+^]_i_ that leads to rapid Ca^2+^ upstroke followed by a plateau-like phase slightly above the baseline. This long-lasting increase in [Ca^2+^]_i_ was evident at concentrations of ML-SA1 ≥ 50 µM. Immunofluorescence and immunoblotting confirmed that the TRPML1 protein was expressed in acidic lysosomal vesicles that were widely distributed throughout the cytosol. These findings demonstrate that the endothelial TRPML1 channel is not only expressed in coronary, but also in brain circulation. Lysosomal-derived Ca^2+^ signals can be amplified into a global increase in [Ca^2+^]_i_ by the recruitment of ER-embedded InsP_3_Rs via the CICR process (Kilpatrick et al., 2013; Penny et al., 2014; Kilpatrick et al., 2016; Galione et al., 2023). An additional pathway that could be activated upon the NAADP-triggered depletion of the InsP_3_-sensitive ER Ca^2+^ store is SOCE, as reported in human cardiac mesenchymal stromal cells (Faris et al., 2022) and MDA-MB-231 cells (Boretto et al., 2023), but not ECFCs (Moccia et al., 2021b) and metastatic colorectal cancer cells (Faris et al., 2019). Furthermore, extracellular Ca^2+^ entry through TRPML1 channels that are located on the plasma membrane can support lysosomal Ca^2+^ mobilization (Kilpatrick et al., 2016). Consistently, in the absence of extracellular Ca^2+^, ML-SA1 evoked a tiny and transient increase in [Ca^2+^]_i_ in hCMEC/D3 cells, which is similar to that recorded under the same conditions in HeLa cells (Kilpatrick et al., 2016). This finding supports the emerging notion that ML-SA1 can induce both intracellular Ca^2+^ release and extracellular Ca^2+^ entry (Kilpatrick et al., 2016; Tedeschi et al., 2021). The intracellular Ca^2+^ response to ML-SA1 was abolished by depleting the lysosomal Ca^2+^ pool and by the pharmacological or genetic blockade of TRPML1. These findings confirm that TRPML1 activation can also result in lysosomal Ca^2+^ mobilization in human cerebrovascular endothelial cells. We must point out that the tiny Ca^2+^ response evoked by ML-SA1 under 0Ca^2+^ conditions was almost suppressed despite the 60% reduction in TRPML1 protein expression achieved by the specific siTRPML1 used in the present study. The following mechanisms could explain these seemingly contradictory results. First, the remaining TRPML1 protein (≈40%) is not expressed in the acidic vesicle membranes and, therefore, cannot contribute to the Ca^2+^ signal. Second, our epifluorescence imaging system does not detect subcellular Ca^2+^ signals, as already reported in (Moccia et al., 2019). Thus, we cannot rule out that some TRPML1-mediated local Ca^2+^ release events still occurred but were not detected. This hypothesis would be further consistent with the evidence that, although lysosomes contain ≈500 µM free Ca^2+^, they occupy ≈3% of the total endothelial cell volume and are sparse throughout the cytoplasm (Lloyd-Evans and Waller-Evans, 2019). Thus, reducing TRPML1 protein expression by 60% could result in discrete subcellular Ca^2+^ signals that can be recorded only with confocal or 2-photon microscopy (Boittin et al., 2002; Kinnear et al., 2008), especially if they are uncoupled from the ER, as further discussed below. For instance, Kinnear et al. revealed that NAADP elicited subcellular Ca^2+^ signals that did not significantly increase Fura-2 fluorescence until they led to ER Ca^2+^ release in rat pulmonary artery vascular smooth muscle cells (Kinnear et al., 2008). In agreement with these hypotheses, we have recently shown that the lysosomal Ca^2+^ response to NAADP is virtually abolished in circulating endothelial colony forming cells transfected with a siRNA selectively targeting TPC1 despite the fact the TPC1 protein expression is downregulated by ≈ 60% (Moccia et al., 2021b). Therefore, we are confident that the overall evidence based upon the pharmacological and genetic blockade of TRPML1 supports its primary role in the Ca^2+^-response to ML-SA1.

ML-SA1-induced intracellular Ca^2+^ mobilization was also attenuated or virtually abolished by depleting the ER Ca^2+^ store and by blocking InsP_3_Rs. These findings are consistent with those reported in HeLa cells (Kilpatrick et al., 2016) and indicate that InsP_3_Rs sustain the lysosomal Ca^2+^ signal in hCMEC/D3 cells. Therefore, the absence of a regenerative Ca^2+^ response at low concentrations of ML-SA1 (<50 µM) can be explained by the failure of spatially-restricted lysosomal Ca^2+^ nanodomains to fully recruit the juxtaposed InsP_3_Rs (Medina et al., 2015; Davis et al., 2023). On the other hand, an increase in the amount of lysosomal Ca^2+^ mobilized by higher concentrations of ML-SA1 (>50 µM) could successfully trigger ER Ca^2+^ release through InsP_3_Rs. Lysosomal Ca^2+^ release could mobilize the ER Ca^2+^ pool by either triggering CICR through RyRs/InsP_3_Rs (Yuan et al., 2024; Yuan et al., 2024) or by refilling the ER in a SERCA-dependent manner, thereby leading to luminal Ca^2+^ overload and InsP_3_R/RyR opening (Macgregor et al., 2007). A recent investigation revealed that TRPML1-mediated lysosomal Ca^2+^ release tonically regulates the ER Ca^2+^ content in primary rat cortical neurons (Tedeschi et al., 2021). Herein, we found that CPA-evoked ER Ca^2+^ release, which can be used as a proxy for the free ER Ca^2+^ concentration (Brandman et al., 2007; Pierro et al., 2014; Lodola et al., 2017), was significantly reduced upon the pharmacological (with ML-SI3) or genetic (with the specific siTRPML1) blockade of TRPML1. This finding strongly suggests that local TRPML1-mediated Ca^2+^ signals are rerouted into the ER in a SERCA-dependent manner, thereby controlling the free ER Ca^2+^ concentration that is available to be released through the ER leakage channels and/or the InsP_3_R. Therefore, it is reasonable to conclude that ML-SA1 gates TRPML1, which is likely to lead to ER Ca^2+^ overload and InsP_3_R activation from the luminal side. Consistent with this hypothesis, the pharmacological blockade of SOCE with two distinct pyrazole derivatives, Pyr6 and BTP-2, converted the global increase in [Ca^2+^]_i_ induced by ML-SA1 into a tiny Ca^2+^ transient that strongly resembled that measured under 0Ca^2+^ conditions. In addition, the “Ca^2+^ add-back” protocol revealed that TRPML1-dependent Ca^2+^ entry did not require the presence of the extracellular agonist to occur, while it was only associated with the previous depletion of the intracellular Ca^2+^ pool. This feature is a hallmark of SOCE activation, which confirms that TRPML1 leads to ER Ca^2+^ depletion (Murata et al., 2007; Bird et al., 2008; Moccia et al., 2023a). Conversely, the Fe^2+^ quench assay revealed that TRPML1 does not directly contribute to extracellular Ca^2+^ entry in hCMEC/D3 cells. A recent study showed that TRPML1 may physically interact with STIM1 (Tedeschi et al., 2021), which could facilitate the assembly of the SOCE machinery. Collectively, these findings strongly suggest that TRPML1-mediated lysosomal Ca^2+^ release can trigger ER Ca^2+^ release through the Ca^2+^-dependent luminal recruitment of InsP_3_Rs, thereby leading to SOCE activation. Nevertheless, a direct measurement of the ER Ca^2+^ concentration is necessary to provide the clear-cut evidence that TRPML1 contributes to ER Ca^2+^ refilling.

The endogenous agonist of TRPML1 is still debated, but several lines of evidence indicate that the lysosomal associated PI(3,5)P_2_ could fulfil this role (Dong et al., 2010; Zhang et al., 2012; Isobe et al., 2019; Li et al., 2019; Negri et al., 2021b). Consistent with this, the membrane permeable analogue of PI(3,5)P_2_ induced a global increase in [Ca^2+^]_i_ that was similar, although slightly slower, to that induced by ML-SA1. Furthermore, the Ca^2+^ response to PI(3,5)P_2_ was inhibited by the depletion of the lysosomal Ca^2+^ store and by the pharmacological or genetic blockade of TRPML1. Therefore, PI(3,5)P_2_ could also serve as an endogenous agonist of TRPML1 in human cerebrovascular endothelial cells, where it could elicit weak or strong Ca^2+^ signals depending on the strength of TRPML1 activation and/or its functional coupling with InsP_3_Rs. A further hint at the physiological role played by TRPML1-mediated global Ca^2+^ signals arises from NO measurements. Single-cell imaging of DAF-FM DA fluorescence showed that ML-SA1 induced robust NO release via the Ca^2+^-dependent recruitment of eNOS. As reported by single-cell Fura-2 imaging, ML-SA1-induced NO production was abolished by preventing lysosomal Ca^2+^ release through TRPML1 and the Ca^2+^-dependent eNOS activation. These findings provide the first evidence that, in addition to NAADP-gated TPCs (Brailoiu et al., 2010; Negri et al., 2021b), the lysosomal Ca^2+^ pool could also lead to endothelium-dependent NO release via TRPML1. A recent study has shown that TRPML1-mediated Ca^2+^ release can trigger Ca^2+^ sparks via CICR through juxtaposed RyRs in vascular smooth muscle cells (VSMCs) from cerebral arteries (Thakore et al., 2020). Ca^2+^ sparks, in turn, induce vasodilation and increase local CBF by activating big conductance Ca^2+^-dependent K^+^ channels (Thakore et al., 2020). However, these data collectively suggest that TRPML1 could be critical to regulate vascular reactivity in brain microcirculation, in both VSMCs and endothelial cells. In addition, TRPML1 could also regulate other functions in human cerebrovascular endothelial cells, including lysosome size and trafficking (Yang et al., 2019), autophagy (Yang et al., 2019), and membrane repair (Morabito et al., 2017; Li et al., 2019). TRPML1 could also be physiologically regulated by reactive oxygen species (ROS) (Zhang et al., 2016), which are emerging as crucial regulators of the endothelial Ca^2+^ signals at the neurovascular unit (Thakore et al., 2021; Berra-Romani et al., 2023). Future work might assess whether ROS modulate TRPML1 activation in human cerebrovascular endothelial cells.

In order to confirm that TRPML1 is involved in agonist-evoked Ca^2+^ signaling in hCMEC/D3 cells, we assessed whether TRPML1 modulates the Ca^2+^ response to ATP, which is one of the most widespread endothelial agonists (Scarpellino et al., 2019; Scarpellino et al., 2022; Lisec et al., 2024). ATP has been shown to elicit InsP_3_-induced ER Ca^2+^ release in hCMEC/D3 cells (Bintig et al., 2012; Forcaia et al., 2021). Herein, we found that ATP-induced Ca^2+^ signals and NO release were both impaired by blocking TRPML1 with ML-SI3 or the specific siTRPML1 or by inhibiting PI(3,5)P_2_ production with apilimod. The most plausible hypothesis to explain these findings is that TRPML1 is gated by PI(3,5)P_2_ to maintain ER Ca^2+^ levels, thereby enabling the proper Ca^2+^ response to ATP.

## 5 Conclusion

The present investigation provides the first evidence that the lysosomal TRPML1 channel is expressed and mediates a global increase in [Ca^2+^]_i_ in human cerebrovascular endothelial cells. Lysosomal Ca^2+^ release through TRPML1 is supported by ER Ca^2+^ mobilization through InsP_3_Rs and by SOCE. Physiologically, TRPML1 could be gated by the lysosome associated phosphoinositide, PI(3,5)P_2_, and could be involved in the regulation of CBF by promoting endothelium-dependent NO release.

## Data Availability

The raw data supporting the conclusion of this article will be made available by the authors, without undue reservation.

## References

[B1] Bader A. Bintig W. Begandt D. Klett A. Siller I. G. Gregor C. (2017). Adenosine receptors regulate gap junction coupling of the human cerebral microvascular endothelial cells hCMEC/D3 by Ca(2+) influx through cyclic nucleotide-gated channels. J. Physiol. 595 (8), 2497–2517. 10.1113/JP273150 28075020 PMC5390872

[B2] Berra-Romani R. Brunetti V. Pellavio G. Soda T. Laforenza U. Scarpellino G. (2023). Allyl isothiocianate induces Ca(2+) signals and nitric oxide release by inducing reactive oxygen species production in the human cerebrovascular endothelial cell line hCMEC/D3. Cells 12 (13), 1732. 10.3390/cells12131732 37443764 PMC10340171

[B3] Berra-Romani R. Faris P. Pellavio G. Orgiu M. Negri S. Forcaia G. (2020). Histamine induces intracellular Ca(2+) oscillations and nitric oxide release in endothelial cells from brain microvascular circulation. J. Cell Physiol. 235 (2), 1515–1530. 10.1002/jcp.29071 31310018

[B4] Bintig W. Begandt D. Schlingmann B. Gerhard L. Pangalos M. Dreyer L. (2012). Purine receptors and Ca(2+) signalling in the human blood-brain barrier endothelial cell line hCMEC/D3. Purinergic Signal 8 (1), 71–80. 10.1007/s11302-011-9262-7 21956217 PMC3286539

[B5] Bird G. S. DeHaven W. I. Smyth J. T. Putney J. W. Jr. (2008). Methods for studying store-operated calcium entry. Methods 46 (3), 204–212. 10.1016/j.ymeth.2008.09.009 18929662 PMC2643845

[B6] Blatter L. A. (2017). Tissue specificity: SOCE: implications for Ca2+ handling in endothelial cells. Adv. Exp. Med. Biol. 993, 343–361. 10.1007/978-3-319-57732-6_18 28900923

[B7] Boittin F. X. Galione A. Evans A. M. (2002). Nicotinic acid adenine dinucleotide phosphate mediates Ca2+ signals and contraction in arterial smooth muscle via a two-pool mechanism. Circ. Res. 91 (12), 1168–1175. 10.1161/01.res.0000047507.22487.85 12480818

[B8] Boretto C. Actis C. Faris P. Cordero F. Beccuti M. Ferrero G. (2023). Tamoxifen activates transcription factor eb and triggers protective autophagy in breast cancer cells by inducing lysosomal calcium release: a gateway to the onset of endocrine resistance. Int. J. Mol. Sci. 25 (1), 458. 10.3390/ijms25010458 38203629 PMC10779225

[B9] Brailoiu G. C. Gurzu B. Gao X. Parkesh R. Aley P. K. Trifa D. I. (2010). Acidic NAADP-sensitive calcium stores in the endothelium: agonist-specific recruitment and role in regulating blood pressure. J. Biol. Chem. 285 (48), 37133–37137. 10.1074/jbc.C110.169763 20876534 PMC2988319

[B10] Brandman O. Liou J. Park W. S. Meyer T. (2007). STIM2 is a feedback regulator that stabilizes basal cytosolic and endoplasmic reticulum Ca2+ levels. Cell 131 (7), 1327–1339. 10.1016/j.cell.2007.11.039 18160041 PMC2680164

[B11] Cao Q. Yang Y. Zhong X. Z. Dong X. P. (2017). The lysosomal Ca(2+) release channel TRPML1 regulates lysosome size by activating calmodulin. J. Biol. Chem. 292 (20), 8424–8435. 10.1074/jbc.M116.772160 28360104 PMC5437247

[B12] Chauvet S. Jarvis L. Chevallet M. Shrestha N. Groschner K. Bouron A. (2016). Pharmacological characterization of the native store-operated calcium channels of cortical neurons from embryonic mouse brain. Front. Pharmacol. 7, 486. 10.3389/fphar.2016.00486 28018223 PMC5149554

[B13] Davis L. C. Morgan A. J. Galione A. (2023). Optical profiling of autonomous Ca(2+) nanodomains generated by lysosomal TPC2 and TRPML1. Cell Calcium 116, 102801. 10.1016/j.ceca.2023.102801 37742482

[B14] De Bock M. Wang N. Decrock E. Bol M. Gadicherla A. K. Culot M. (2013). Endothelial calcium dynamics, connexin channels and blood-brain barrier function. Prog. Neurobiol. 108, 1–20. 10.1016/j.pneurobio.2013.06.001 23851106

[B15] Di Nezza F. Zuccolo E. Poletto V. Rosti V. De Luca A. Moccia F. (2017). Liposomes as a putative tool to investigate NAADP signaling in vasculogenesis. J. Cell Biochem. 118 (11), 3722–3729. 10.1002/jcb.26019 28374913

[B16] Dong X. P. Shen D. Wang X. Dawson T. Li X. Zhang Q. (2010). PI(3,5)P(2) controls membrane trafficking by direct activation of mucolipin Ca(2+) release channels in the endolysosome. Nat. Commun. 1, 38. 10.1038/ncomms1037 20802798 PMC2928581

[B17] Esposito B. Gambara G. Lewis A. M. Palombi F. D’Alessio A. Taylor L. X. (2011). NAADP links histamine H1 receptors to secretion of von Willebrand factor in human endothelial cells. Blood 117 (18), 4968–4977. 10.1182/blood-2010-02-266338 21364192

[B18] Faris P. Casali C. Negri S. Iengo L. Biggiogera M. Maione A. S. (2022). Nicotinic acid adenine dinucleotide phosphate induces intracellular Ca(2+) signalling and stimulates proliferation in human cardiac mesenchymal stromal cells. Front. Cell Dev. Biol. 10, 874043. 10.3389/fcell.2022.874043 35392169 PMC8980055

[B19] Faris P. Pellavio G. Ferulli F. Di Nezza F. Shekha M. Lim D. (2019). Nicotinic acid adenine dinucleotide phosphate (NAADP) induces intracellular Ca(2+) release through the two-pore channel TPC1 in metastatic colorectal cancer cells. Cancers (Basel) 11 (4), 542. 10.3390/cancers11040542 30991693 PMC6521149

[B20] Favia A. Desideri M. Gambara G. D’Alessio A. Ruas M. Esposito B. (2014). VEGF-induced neoangiogenesis is mediated by NAADP and two-pore channel-2-dependent Ca2+ signaling. Proc. Natl. Acad. Sci. U. S. A. 111 (44), E4706–E4715. 10.1073/pnas.1406029111 25331892 PMC4226099

[B21] Forcaia G. Formicola B. Terribile G. Negri S. Lim D. Biella G. (2021). Multifunctional liposomes modulate purinergic receptor-induced calcium wave in cerebral microvascular endothelial cells and astrocytes: new insights for alzheimer’s disease. Mol. Neurobiol. 58 (6), 2824–2835. 10.1007/s12035-021-02299-9 33511502 PMC8128821

[B22] Galione A. Davis L. C. Martucci L. L. Morgan A. J. (2023). NAADP-mediated Ca(2+) signalling. Handb. Exp. Pharmacol. 278, 3–34. 10.1007/164_2022_607 35879580

[B95] Garrity A. G. Wang W. Collier C. M. D. Levey S. A. Gao Q. Xu H. (2016). The endoplasmic reticulum, not the pH gradient, drives calcium refilling of lysosomes. eLife (5), e15887. 10.7554/eLife 27213518 PMC4909396

[B23] Greenberg H. Z. E. Carlton-Carew S. R. E. Khan D. M. Zargaran A. K. Jahan K. S. Vanessa Ho W. S. (2017). Heteromeric TRPV4/TRPC1 channels mediate calcium-sensing receptor-induced nitric oxide production and vasorelaxation in rabbit mesenteric arteries. Vasc. Pharmacol. 96-98, 53–62. 10.1016/j.vph.2017.08.005 PMC5614111 28867591

[B24] Groschner K. Shrestha N. Fameli N. (2017). Cardiovascular and hemostatic disorders: SOCE in cardiovascular cells: emerging targets for therapeutic intervention. Adv. Exp. Med. Biol. 993, 473–503. 10.1007/978-3-319-57732-6_24 28900929

[B25] Helms H. C. Abbott N. J. Burek M. Cecchelli R. Couraud P. O. Deli M. A. (2016). *In vitro* models of the blood-brain barrier: an overview of commonly used brain endothelial cell culture models and guidelines for their use. J. Cereb. Blood Flow. Metab. 36 (5), 862–890. 10.1177/0271678X16630991 26868179 PMC4853841

[B26] Hou Y. He H. Ma M. Zhou R. (2023). Apilimod activates the NLRP3 inflammasome through lysosome-mediated mitochondrial damage. Front. Immunol. 14, 1128700. 10.3389/fimmu.2023.1128700 37359517 PMC10285205

[B27] Isobe Y. Nigorikawa K. Tsurumi G. Takemasu S. Takasuga S. Kofuji S. (2019). PIKfyve accelerates phagosome acidification through activation of TRPML1 while arrests aberrant vacuolation independent of the Ca2+ channel. J. Biochem. 165 (1), 75–84. 10.1093/jb/mvy084 30295876

[B28] Kilpatrick B. S. Eden E. R. Schapira A. H. Futter C. E. Patel S. (2013). Direct mobilisation of lysosomal Ca2+ triggers complex Ca2+ signals. J. Cell Sci. 126 (Pt 1), 60–66. 10.1242/jcs.118836 23108667 PMC4208295

[B29] Kilpatrick B. S. Yates E. Grimm C. Schapira A. H. Patel S. (2016). Endo-lysosomal TRP mucolipin-1 channels trigger global ER Ca2+ release and Ca2+ influx. J. Cell Sci. 129 (20), 3859–3867. 10.1242/jcs.190322 27577094 PMC5087663

[B30] Kinnear N. P. Wyatt C. N. Clark J. H. Calcraft P. J. Fleischer S. Jeyakumar L. H. (2008). Lysosomes co-localize with ryanodine receptor subtype 3 to form a trigger zone for calcium signalling by NAADP in rat pulmonary arterial smooth muscle. Cell Calcium 44 (2), 190–201. 10.1016/j.ceca.2007.11.003 18191199 PMC3982125

[B31] Kuppusamy M. Ottolini M. Sonkusare S. K. (2021). Role of TRP ion channels in cerebral circulation and neurovascular communication. Neurosci. Lett. 765, 136258. 10.1016/j.neulet.2021.136258 34560190 PMC8572163

[B32] Lai Y. Yang N. Shi D. Ma X. Huang Y. Lu J. (2024). Puerarin enhances TFEB-mediated autophagy and attenuates ROS-induced pyroptosis after ischemic injury of random-pattern skin flaps. Eur. J. Pharmacol. 974, 176621. 10.1016/j.ejphar.2024.176621 38679118

[B33] Li G. Huang D. Li P. Yuan X. Yarotskyy V. Li P. L. (2022). Regulation of exosome release by lysosomal acid ceramidase in coronary arterial endothelial cells: role of TRPML1 channel. Curr. Top. Membr. 90, 37–63. 10.1016/bs.ctm.2022.09.002 36368874 PMC9842397

[B34] Li P. Gu M. Xu H. (2019). Lysosomal ion channels as decoders of cellular signals. Trends Biochem. Sci. 44 (2), 110–124. 10.1016/j.tibs.2018.10.006 30424907 PMC6340733

[B35] Lisec B. Bozic T. Santek I. Markelc B. Vrecl M. Frangez R. (2024). Characterization of two distinct immortalized endothelial cell lines, EA.hy926 and HMEC-1, for *in vitro* studies: exploring the impact of calcium electroporation, Ca^2+^ signaling and transcriptomic profiles. Cell Commun. Signal 22 (1), 118. 10.1186/s12964-024-01503-2 38347539 PMC10863159

[B36] Lloyd-Evans E. Waller-Evans H. (2019). Lysosomal Ca(2+) homeostasis and signaling in health and disease. Cold Spring Harb. Perspect. Biol. 12, a035311. 10.1101/cshperspect.a035311 PMC7263086 31653642

[B37] Lodola F. Laforenza U. Cattaneo F. Ruffinatti F. A. Poletto V. Massa M. (2017). VEGF-induced intracellular Ca^2+^ oscillations are down-regulated and do not stimulate angiogenesis in breast cancer-derived endothelial colony forming cells. Oncotarget 8, 95223–95246. 10.18632/oncotarget.20255 29221123 PMC5707017

[B38] Longden T. A. Mughal A. Hennig G. W. Harraz O. F. Shui B. Lee F. K. (2021). Local IP3 receptor-mediated Ca(2+) signals compound to direct blood flow in brain capillaries. Sci. Adv. 7 (30), eabh0101. 10.1126/sciadv.abh0101 34290098 PMC8294755

[B39] Luo H. Rossi E. Saubamea B. Chasseigneaux S. Cochois V. Choublier N. (2019). Cannabidiol increases proliferation, migration, tubulogenesis, and integrity of human brain endothelial cells through TRPV2 activation. Mol. Pharm. 16 (3), 1312–1326. 10.1021/acs.molpharmaceut.8b01252 30721081

[B40] Macgregor A. Yamasaki M. Rakovic S. Sanders L. Parkesh R. Churchill G. C. (2007). NAADP controls cross-talk between distinct Ca2+ stores in the heart. J. Biol. Chem. 282 (20), 15302–15311. 10.1074/jbc.M611167200 17387177

[B41] McCarron J. G. Lee M. D. Wilson C. (2017). The endothelium solves problems that endothelial cells do not know exist. Trends Pharmacol. Sci. 38 (4), 322–338. 10.1016/j.tips.2017.01.008 28214012 PMC5381697

[B42] Medina D. L. Di Paola S. Peluso I. Armani A. De Stefani D. Venditti R. (2015). Lysosomal calcium signalling regulates autophagy through calcineurin and TFEB. Nat. Cell Biol. 17 (3), 288–299. 10.1038/ncb3114 25720963 PMC4801004

[B43] Missiaen L. Taylor C. W. Berridge M. J. (1992). Luminal Ca2+ promoting spontaneous Ca2+ release from inositol trisphosphate-sensitive stores in rat hepatocytes. J. Physiol. 455, 623–640. 10.1113/jphysiol.1992.sp019319 1484365 PMC1175662

[B44] Moccia F. Brunetti V. Perna A. Guerra G. Soda T. Berra-Romani R. (2023a). The molecular heterogeneity of store-operated Ca(2+) entry in vascular endothelial cells: the different roles of Orai1 and TRPC1/TRPC4 channels in the transition from Ca(2+)-selective to non-selective cation currents. Int. J. Mol. Sci. 24 (4), 3259. 10.3390/ijms24043259 36834672 PMC9967124

[B45] Moccia F. Brunetti V. Soda T. Berra-Romani R. Scarpellino G. (2023b). Cracking the endothelial calcium (Ca(2+)) code: a matter of timing and spacing. Int. J. Mol. Sci. 24 (23), 16765. 10.3390/ijms242316765 38069089 PMC10706333

[B46] Moccia F. Fiorio Pla A. Lim D. Lodola F. Gerbino A. (2023c). Intracellular Ca(2+) signalling: unexpected new roles for the usual suspect. Front. Physiol. 14, 1210085. 10.3389/fphys.2023.1210085 37576340 PMC10413985

[B47] Moccia F. Negri S. Faris P. Angelone T. (2022). Targeting endothelial ion signalling to rescue cerebral blood flow in cerebral disorders. Vasc. Pharmacol. 145, 106997. 10.1016/j.vph.2022.106997 35526818

[B48] Moccia F. Negri S. Faris P. Perna A. De Luca A. Soda T. (2021a). Targeting endolysosomal two-pore channels to treat cardiovascular disorders in the novel COronaVIrus disease 2019. Front. Physiol. 12, 629119. 10.3389/fphys.2021.629119 33574769 PMC7870486

[B49] Moccia F. Negri S. Shekha M. Faris P. Guerra G. (2019). Endothelial Ca(2+) signaling, angiogenesis and vasculogenesis: just what it takes to make a blood vessel. Int. J. Mol. Sci. 20 (16), 3962. 10.3390/ijms20163962 31416282 PMC6721072

[B50] Moccia F. Zuccolo E. Di Nezza F. Pellavio G. Faris P. S. Negri S. (2021b). Nicotinic acid adenine dinucleotide phosphate activates two-pore channel TPC1 to mediate lysosomal Ca(2+) release in endothelial colony-forming cells. J. Cell Physiol. 236 (1), 688–705. 10.1002/jcp.29896 32583526

[B51] Morabito R. Costa R. Rizzo V. Remigante A. Nofziger C. La Spada G. (2017). Crude venom from nematocysts of Pelagia noctiluca (Cnidaria: scyphozoa) elicits a sodium conductance in the plasma membrane of mammalian cells. Sci. Rep. 7, 41065. 10.1038/srep41065 28112211 PMC5253680

[B52] Morgan A. J. (2016). Ca2+ dialogue between acidic vesicles and ER. Biochem. Soc. Trans. 44 (2), 546–553. 10.1042/BST20150290 27068968

[B53] Morgan A. J. Galione A. (2021). Lysosomal agents inhibit store-operated Ca(2+) entry. J. Cell Sci. 134 (2), jcs248658. 10.1242/jcs.248658 33328326 PMC7860125

[B54] Morgan A. J. Platt F. M. Lloyd-Evans E. Galione A. (2011). Molecular mechanisms of endolysosomal Ca2+ signalling in health and disease. Biochem. J. 439 (3), 349–374. 10.1042/BJ20110949 21992097

[B55] Mughal A. Sackheim A. M. Koide M. Bonson G. Ebner G. Hennig G. (2024). Pathogenic soluble tau peptide disrupts endothelial calcium signaling and vasodilation in the brain microvasculature. J. Cereb. Blood Flow. Metab. 271678X241235790, 680–688. 10.1177/0271678X241235790 PMC11197144 38420777

[B56] Murata T. Lin M. I. Stan R. V. Bauer P. M. Yu J. Sessa W. C. (2007). Genetic evidence supporting caveolae microdomain regulation of calcium entry in endothelial cells. J. Biol. Chem. 282 (22), 16631–16643. 10.1074/jbc.M607948200 17416589

[B57] Mussano F. Genova T. Laurenti M. Gaglioti D. Scarpellino G. Rivolo P. (2020). Beta1-integrin and TRPV4 are involved in osteoblast adhesion to different titanium surface topographies. Appl. Surf. Sci. 507, 145112. 10.1016/j.apsusc.2019.145112

[B58] Negri S. Faris P. Berra-Romani R. Guerra G. Moccia F. (2019). Endothelial transient receptor potential channels and vascular remodeling: extracellular Ca(2 +) entry for angiogenesis, arteriogenesis and vasculogenesis. Front. Physiol. 10, 1618. 10.3389/fphys.2019.01618 32038296 PMC6985578

[B59] Negri S. Faris P. Maniezzi C. Pellavio G. Spaiardi P. Botta L. (2021a). NMDA receptors elicit flux-independent intracellular Ca(2+) signals via metabotropic glutamate receptors and flux-dependent nitric oxide release in human brain microvascular endothelial cells. Cell Calcium 99, 102454. 10.1016/j.ceca.2021.102454 34454368

[B60] Negri S. Faris P. Moccia F. (2021b). Endolysosomal Ca(2+) signaling in cardiovascular health and disease. Int. Rev. Cell Mol. Biol. 363, 203–269. 10.1016/bs.ircmb.2021.03.001 34392930

[B61] Negri S. Faris P. Pellavio G. Botta L. Orgiu M. Forcaia G. (2020). Group 1 metabotropic glutamate receptors trigger glutamate-induced intracellular Ca(2+) signals and nitric oxide release in human brain microvascular endothelial cells. Cell Mol. Life Sci. 77 (11), 2235–2253. 10.1007/s00018-019-03284-1 31473770 PMC11104941

[B62] Negri S. Faris P. Soda T. Moccia F. (2021c). Endothelial signaling at the core of neurovascular coupling: the emerging role of endothelial inward-rectifier K(+) (Kir2.1) channels and N-methyl-d-aspartate receptors in the regulation of cerebral blood flow. Int. J. Biochem. Cell Biol. 135, 105983. 10.1016/j.biocel.2021.105983 33894355

[B63] Negri S. Scolari F. Vismara M. Brunetti V. Faris P. Terribile G. (2022). GABA(A) and GABA(B) receptors mediate GABA-induced intracellular Ca(2+) signals in human brain microvascular endothelial cells. Cells 11 (23), 3860. 10.3390/cells11233860 36497118 PMC9739010

[B64] Ottolini M. Hong K. Sonkusare S. K. (2019). Calcium signals that determine vascular resistance. Wiley Interdiscip. Rev. Syst. Biol. Med. 11 (5), e1448. 10.1002/wsbm.1448 30884210 PMC6688910

[B65] Pafumi I. Favia A. Gambara G. Papacci F. Ziparo E. Palombi F. (2015). Regulation of angiogenic functions by angiopoietins through calcium-dependent signaling pathways. Biomed. Res. Int. 2015, 965271. 10.1155/2015/965271 26146638 PMC4471310

[B66] Penny C. J. Kilpatrick B. S. Han J. M. Sneyd J. Patel S. (2014). A computational model of lysosome-ER Ca2+ microdomains. J. Cell Sci. 127 (Pt 13), 2934–2943. 10.1242/jcs.149047 24706947

[B67] Peters E. C. Gee M. T. Pawlowski L. N. Kath A. M. Polk F. D. Vance C. J. (2022). Amyloid-*β* disrupts unitary calcium entry through endothelial NMDA receptors in mouse cerebral arteries. J. Cereb. Blood Flow. Metab. 42 (1), 145–161. 10.1177/0271678X211039592 34465229 PMC8721780

[B68] Pierro C. Cook S. J. Foets T. C. Bootman M. D. Roderick H. L. (2014). Oncogenic K-Ras suppresses IP₃-dependent Ca^2^⁺ release through remodelling of the isoform composition of IP₃Rs and ER luminal Ca^2^⁺ levels in colorectal cancer cell lines. J. Cell Sci. 127 (Pt 7), 1607–1619. 10.1242/jcs.141408 24522186

[B69] Riva B. Dionisi M. Potenzieri A. Chiorazzi A. Cordero-Sanchez C. Rigolio R. (2018). Oxaliplatin induces pH acidification in dorsal root ganglia neurons. Sci. Rep. 8 (1), 15084. 10.1038/s41598-018-33508-6 30305703 PMC6180129

[B70] Scarpellino G. Genova T. Avanzato D. Bernardini M. Bianco S. Petrillo S. (2019). Purinergic calcium signals in tumor-derived endothelium. Cancers (Basel) 11 (6), 766. 10.3390/cancers11060766 31159426 PMC6627696

[B71] Scarpellino G. Genova T. Quarta E. Distasi C. Dionisi M. Fiorio Pla A. (2022). P2X purinergic receptors are multisensory detectors for micro-environmental stimuli that control migration of tumoral endothelium. Cancers (Basel) 14 (11), 2743. 10.3390/cancers14112743 35681724 PMC9179260

[B72] Schleifer H. Doleschal B. Lichtenegger M. Oppenrieder R. Derler I. Frischauf I. (2012). Novel pyrazole compounds for pharmacological discrimination between receptor-operated and store-operated Ca(2+) entry pathways. Br. J. Pharmacol. 167 (8), 1712–1722. 10.1111/j.1476-5381.2012.02126.x 22862290 PMC3525873

[B73] Scorza S. I. Milano S. Saponara I. Certini M. De Zio R. Mola M. G. (2023). TRPML1-Induced lysosomal Ca(2+) signals activate AQP2 translocation and water flux in renal collecting duct cells. Int. J. Mol. Sci. 24 (2), 1647. 10.3390/ijms24021647 36675161 PMC9861594

[B74] Sheng J. Z. Braun A. P. (2007). Small- and intermediate-conductance Ca2+-activated K+ channels directly control agonist-evoked nitric oxide synthesis in human vascular endothelial cells. Am. J. Physiol. Cell Physiol. 293 (1), C458–C467. 10.1152/ajpcell.00036.2007 17459950

[B75] Sheng J. Z. Wang D. Braun A. P. (2005). DAF-FM (4-amino-5-methylamino-2’,7'-difluorofluorescein) diacetate detects impairment of agonist-stimulated nitric oxide synthesis by elevated glucose in human vascular endothelial cells: reversal by vitamin C and L-sepiapterin. J. Pharmacol. Exp. Ther. 315 (2), 931–940. 10.1124/jpet.105.087932 16093274

[B76] Soda T. Brunetti V. Berra-Romani R. Moccia F. (2023). The emerging role of N-Methyl-D-Aspartate (NMDA) receptors in the cardiovascular system: physiological implications, pathological consequences, and therapeutic perspectives. Int. J. Mol. Sci. 24 (4), 3914. 10.3390/ijms24043914 36835323 PMC9965111

[B77] Somogyi A. Kirkham E. D. Lloyd-Evans E. Winston J. Allen N. D. Mackrill J. J. (2023). The synthetic TRPML1 agonist ML-SA1 rescues Alzheimer-related alterations of the endosomal-autophagic-lysosomal system. J. Cell Sci. 136 (6), jcs259875. 10.1242/jcs.259875 36825945 PMC10112969

[B78] Tedeschi V. Sisalli M. J. Petrozziello T. Canzoniero L. M. T. Secondo A. (2021). Lysosomal calcium is modulated by STIM1/TRPML1 interaction which participates to neuronal survival during ischemic preconditioning. FASEB J. 35 (2), e21277. 10.1096/fj.202001886R 33484198

[B79] Thakore P. Alvarado M. G. Ali S. Mughal A. Pires P. W. Yamasaki E. (2021). Brain endothelial cell TRPA1 channels initiate neurovascular coupling. Elife 10, e63040. 10.7554/eLife.63040 33635784 PMC7935492

[B80] Thakore P. Pritchard H. A. T. Griffin C. S. Yamasaki E. Drumm B. T. Lane C. (2020). TRPML1 channels initiate Ca(2+) sparks in vascular smooth muscle cells. Sci. Signal 13 (637), eaba1015. 10.1126/scisignal.aba1015 32576680 PMC7397860

[B81] Wang W. Gao Q. Yang M. Zhang X. Yu L. Lawas M. (2015). Up-regulation of lysosomal TRPML1 channels is essential for lysosomal adaptation to nutrient starvation. Proc. Natl. Acad. Sci. U. S. A. 112 (11), E1373–E1381. 10.1073/pnas.1419669112 25733853 PMC4371935

[B82] Weksler B. Romero I. A. Couraud P. O. (2013). The hCMEC/D3 cell line as a model of the human blood brain barrier. Fluids Barriers CNS 10 (1), 16. 10.1186/2045-8118-10-16 23531482 PMC3623852

[B83] Xu H. Ren D. (2015). Lysosomal physiology. Annu. Rev. Physiol. 77, 57–80. 10.1146/annurev-physiol-021014-071649 25668017 PMC4524569

[B84] Yang N. Yu G. Lai Y. Zhao J. Chen Z. Chen L. (2024). A snake cathelicidin enhances transcription factor EB-mediated autophagy and alleviates ROS-induced pyroptosis after ischaemia-reperfusion injury of island skin flaps. Br. J. Pharmacol. 181 (7), 1068–1090. 10.1111/bph.16268 37850255

[B85] Yang Y. Xu M. Zhu X. Yao J. Shen B. Dong X. P. (2019). Lysosomal Ca^2+^ release channel TRPML1 regulates lysosome size by promoting mTORC1 activity. Eur. J. Cell Biol. 98 (2-4), 116–123. 10.1016/j.ejcb.2019.05.001 31122790

[B86] Yuan Y. Arige V. Saito R. Mu Q. Brailoiu G. C. Pereira G. J. S. (2024). Two-pore channel-2 and inositol trisphosphate receptors coordinate Ca^2+^ signals between lysosomes and the endoplasmic reticulum. Cell Rep. 43 (1), 113628. 10.1016/j.celrep.2023.113628 38160394 PMC10931537

[B87] Zhang M. Mao C. Dai Y. Xu X. Wang X. (2024). Qixian granule inhibits ferroptosis in vascular endothelial cells by modulating TRPML1 in the lysosome to prevent postmenopausal atherosclerosis. J. Ethnopharmacol. 28 (328), 118076. 10.1016/j.jep.2024.118076 38521431

[B88] Zhang X. Cheng X. Yu L. Yang J. Calvo R. Patnaik S. (2016). MCOLN1 is a ROS sensor in lysosomes that regulates autophagy. Nat. Commun. 7, 12109. 10.1038/ncomms12109 27357649 PMC4931332

[B89] Zhang X. Li X. Xu H. (2012). Phosphoinositide isoforms determine compartment-specific ion channel activity. Proc. Natl. Acad. Sci. U. S. A. 109 (28), 11384–11389. 10.1073/pnas.1202194109 22733759 PMC3396495

[B90] Zhang X. Xin P. Yoast R. E. Emrich S. M. Johnson M. T. Pathak T. (2020). Distinct pharmacological profiles of ORAI1, ORAI2, and ORAI3 channels. Cell Calcium 91, 102281. 10.1016/j.ceca.2020.102281 32896813 PMC7654283

[B91] Zuccolo E. Bottino C. Diofano F. Poletto V. Codazzi A. C. Mannarino S. (2016). Constitutive store-operated Ca(2+) entry leads to enhanced nitric oxide production and proliferation in infantile hemangioma-derived endothelial colony-forming cells. Stem Cells Dev. 25 (4), 301–319. 10.1089/scd.2015.0240 26654173

[B92] Zuccolo E. Di Buduo C. Lodola F. Orecchioni S. Scarpellino G. Kheder D. A. (2018). Stromal cell-derived factor-1α promotes endothelial colony-forming cell migration through the Ca2+-dependent activation of the extracellular signal-regulated kinase 1/2 and phosphoinositide 3-kinase/AKT pathways. Stem Cells Dev. 27 (1), 23–34. 10.1089/scd.2017.0114 29121817

[B93] Zuccolo E. Kheder D. A. Lim D. Perna A. Nezza F. D. Botta L. (2019a). Glutamate triggers intracellular Ca(2+) oscillations and nitric oxide release by inducing NAADP- and InsP3 -dependent Ca(2+) release in mouse brain endothelial cells. J. Cell Physiol. 234 (4), 3538–3554. 10.1002/jcp.26953 30451297

[B94] Zuccolo E. Laforenza U. Negri S. Botta L. Berra-Romani R. Faris P. (2019b). Muscarinic M5 receptors trigger acetylcholine-induced Ca(2+) signals and nitric oxide release in human brain microvascular endothelial cells. J. Cell Physiol. 234 (4), 4540–4562. 10.1002/jcp.27234 30191989

