# Supplementary material for: Lysosomal TRPML1 triggers global Ca2+ signals and nitric oxide release in human cerebrovascular endothelial cells
Source: Front Physiol. 2024 Jun 21;15:1426783. doi: 10.3389/fphys.2024.1426783 (PMC11224436; doi:10.3389/fphys.2024.1426783)
Supplement: Supplementary file 1 [file DataSheet1.docx]

Supplementary Material

Lysosomal TRPML1 triggers global Ca^2+^ signals and nitric oxide release in human cerebrovascular endothelial cells

**Valentina Brunetti, Roberto Berra-Romani, Filippo Conca, Teresa Soda, Gerardo Biella, Andrea Gerbino, Francesco Moccia^*^, Giorgia Scarpellino**

*** Correspondence:**Francesco Moccia
[francesco.moccia@unimol.it](mailto:francesco.moccia@unimol.it)

#
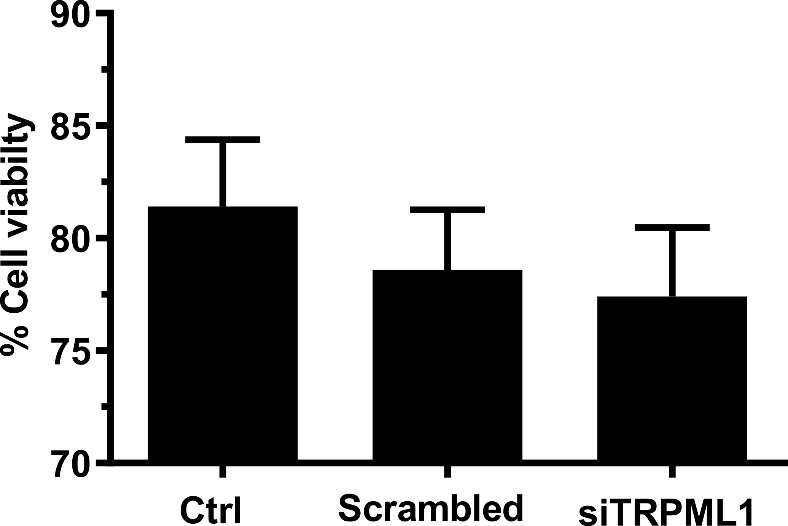
Supplementary Figures

**Supplementary Figure 1.** The Trypan blue exclusion assay showed that hCMEC/D3 cell viability was not affected by a scrambled siRNA (scrambled) or the specific siTRPML1 at 48 hours from the transfection.


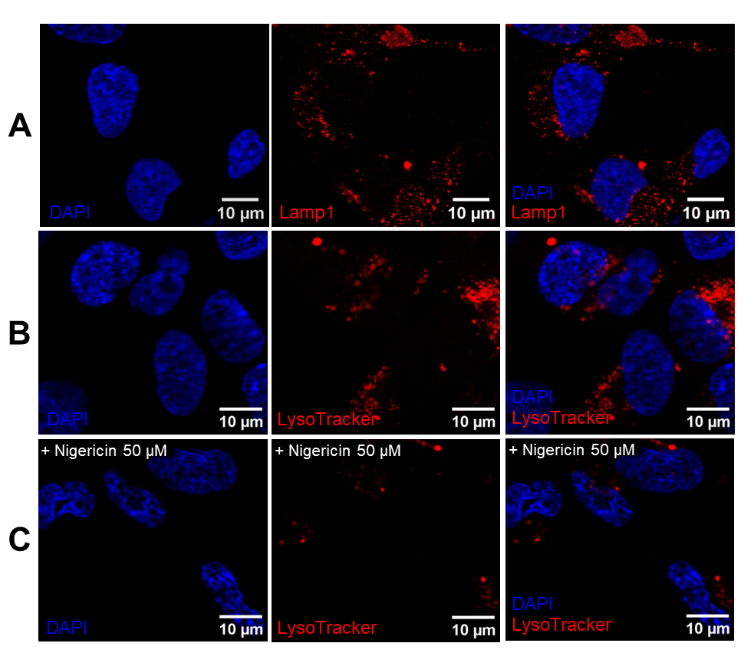


**Supplementary Figure 2.** **(A)** Representative confocal fluorescence image of hCMEC/D3 cells loaded with the lysosomal marker LAMP-1 and **(B)** LysoTracker-Red DND-99 to mark acidic organelles. Nuclei were stained using DAPI (blue). Scale bar: 10 µm. **(C)** Representative confocal fluorescence images of hCMEC/D3 cells pre-incubated with 50 µM Nigericin , reflecting the depletion of lysosomal Ca^2+^ stores. Acidic organelles were marked with LysoTracker-Red DND-99 (red) and nuclei were stained using DAPI (blue). Scale bar: 10 µm.


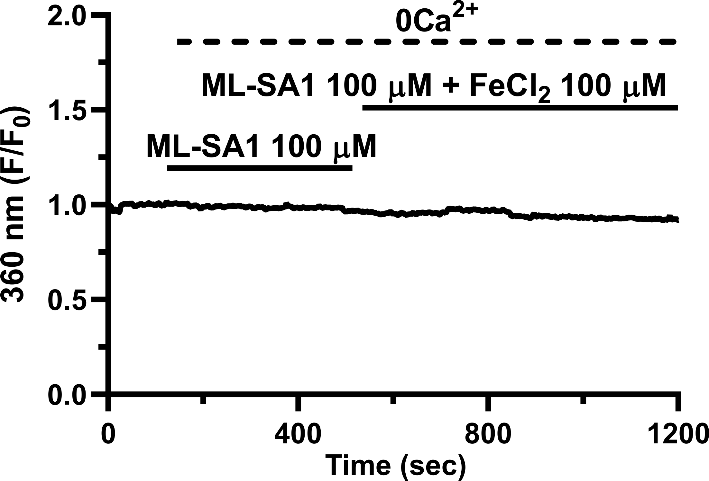


**Supplementary Figure 3.** TRPML1 does not mediate Ca^2+^ influx in hCMEC/D3 cells. The extracellular application of FeCl_2_ (100 μM) did not induce any detectable quench in Fura-2 fluorescence challenged with ML-SA1 (100 μM), suggesting that TRPML1 does not directly contribute to ML-SA1-evoked Ca^2+^ entry. The graph shows the relative fluorescence (F/F_0_) of Fura-2 at 360 nm, i.e. the isosbestic point of Fura-2, where F is the fluorescence intensity obtained during recordings and F_0_ is the basal fluorescence intensity. The tracing is representative of 120 cells recorded from three independent experiments.


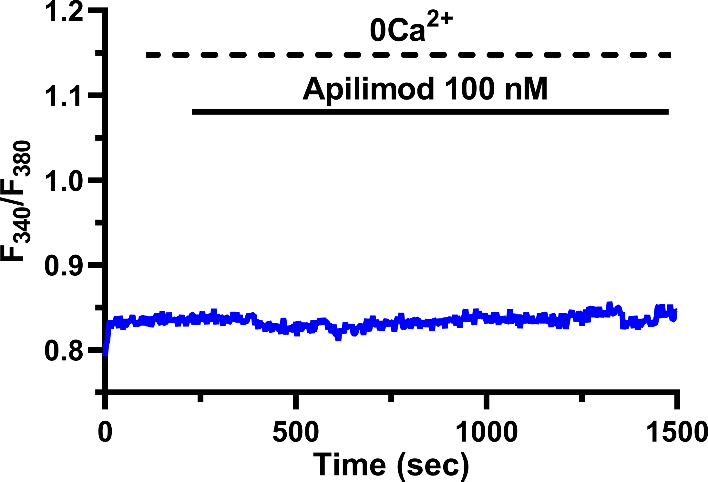


**Supplementary Figure 4.** Apilimod does not increase the [Ca^2+^]_i_ in hCMEC/D3 cells. The treatment with 100 nM Apilimod in the absence of extracellular Ca^2+^ (0Ca^2+^) did not induce any detectable Ca^2+^ signal in hCMEC/D3 cells. The tracing is representative of 96 cells recorded from three independent experiments.
